# Antimicrobial Resistance and Environmental Health: A Water Stewardship Framework for Global and National Action

**DOI:** 10.3390/antibiotics11010063

**Published:** 2022-01-05

**Authors:** Rachel A. Kaiser, Lina Taing, Himesh Bhatia

**Affiliations:** 1 School of Environmental Studies, College of Interdisciplinary Studies, Tennessee Technological University, 1 William L Jones Drive, Cookeville, TN 38505, USA; 2 United Nations University Institute for Water, Environment and Health (UNU-INWEH), 204-175 Longwood Rd. S, Hamilton, ON L8P 0A1, Canada; lina.taing@unu.edu (L.T.); himesh.bhatia@queensu.ca (H.B.); 3 School of Geography and Earth Sciences, McMaster University, 1280 Main Street West, Hamilton, ON L8S 4K1, Canada

**Keywords:** antimicrobial resistance (AMR), one health, environmental health, water stewardship, integrated water resource management (IWRM), water, sanitation and hygiene (WASH)

## Abstract

Antimicrobial resistance (AMR) is a global health crisis that affects all life on Earth. In 2015, the World Health Organization developed guidance to combat AMR in accordance with a One Health framework considering human, animal, and environment sectors of planetary health. This study reviewed global guidance and 25 National Action Plans to evaluate thematic priorities in One Health AMR approaches using a novel framework that additionally facilitated the identification of water-related stewardship gaps, as water resources are recognized as the primary environmental AMR reservoir and dissemination pathway. This review found that global and national stewardship primarily focuses on mitigating antibiotic use in the human and animal sectors, overlooking environmental drivers, particularly diverse environmental waters. The findings of this study highlight the need to broaden the scope of water-related AMR concerns beyond water, sanitation, and hygiene (WASH) infrastructure for water supply and wastewater treatment, and account for environmental waters in AMR development and dissemination, particularly in low-income countries where half a billion people rely on environmental waters to meet daily needs. Equitably accounting for water environments, supplies, and waste in AMR prevention, mitigation, surveillance, and innovation can significantly enhance the integration of environmental objectives in One Health AMR stewardship.

## 1. Introduction

Antibiotic resistance is a natural evolutionary process in microbials, but the improper and overuse of antibiotics are accelerating this process and threatening human health and well-being. The global consumption of antibiotics for human use increased 65% from 2000 to 2015 due to increased access and improper use, particularly in low-income countries (LICs) and middle-income countries (MICs) where health systems are more fragile and rates of improper use are higher [1]. Increased antimicrobial resistance (AMR) exposure from animals and food supplies further exacerbates human AMR risk. The use of antimicrobials in animals for food production is nearly triple the use in humans due to the dual objectives of reducing infection and promoting growth and is projected to increase to 200,235 tons used in animals and 13,600 tons used in aquaculture annually by 2030 [1].

As a result of anthropogenic contamination, AMR organisms have especially been detected in environmental soils and waters, including surface and groundwater [2,3,4,5,6,7,8,9], as a result of antibiotic use in agriculture, aquaculture, pharmaceutical production, and human use. Evidence indicates that wastewater treatment plants are also significant reservoirs and dissemination pathways for AMR [10,11,12,13,14] due to the mixing of bacteria, antibiotics, and other pollutants such as heavy metals, which increases the potential development of AMR organisms [15,16,17,18,19,20]. While there is limited data concerning environmental and water-related AMR risks, up to 90% of antimicrobial doses can be excreted as an active compound or metabolites into the environment [21]. The environment hence is also considered a major AMR reservoir and dissemination pathway given human exposure through direct consumption of food products and water supplies [22,23], and exposure through contaminated soil and the broader environment [24].

As a result of increased human AMR, 700,000 people die annually due to drug-resistant microorganisms, which is predicted to increase to 10 million annual deaths by 2050 if no measures are undertaken to address this global health threat [25]. A cumulative USD 100 trillion of global economic output by 2050, including loss of productivity, is at risk because of AMR [26]. Given AMR’s substantial impact on society, ranging from increased healthcare cost to economic losses, the World Health Organization (WHO) in 2015 declared AMR a global health crisis that needs to be managed to protect global human health and maintain societal scientific and development advancements [27].

In 2015, the WHO established the Global Action Plan on Antimicrobial Resistance (GAP) to mitigate antibacterial resistance through a holistic One Health approach for AMR in humans, animals, and the environment [28]. The One Health conceptual framework considers interconnections between humans, animals, and the environment in which resistance can cross social, ecological, and habitat boundaries [29]. The five main objectives of the WHO’s GAP are: (1) improving awareness and understanding, (2) strengthening surveillance and research, (3) reducing the incidence of infection, (4) optimizing the use of antimicrobial medicines, and (5) ensuring sustainable investment in countering resistance [28].

In response to the WHO’s call, the United Nations (UN) established the One Health Tripartite in 2016, tasking the intergovernmental organizations the WHO, the Food and Agriculture Organization (FAO), and the World Organization for Animal Health (OIE) to address emerging global AMR risks through a One Health approach [30]. The United Nations Interagency Coordination Group on Antimicrobial Resistance (IACG) is also active in achieving the GAP objectives through greater integration of AMR activities in the Sustainable Development Goal (SDG) agenda [25,31]. These intergovernmental entities have developed a variety of policy instruments that provide an evolving stewardship framework for combating AMR holistically. The global instruments—ranging from guidelines, tools, and roadmaps—support achievement of the GAP objectives at the national level through development and implementation of National Action Plans (NAP).

Despite the holistic One Health lens, the primary focus of these global instruments has been the human and animal sectors, mainly through antimicrobial consumption and human infection prevention and control (IPC), leaving the environment sector under-supported at the global and, in turn, the national levels [32,33,34]. Some guidelines, tools, and roadmaps loosely incorporate the environment, including water-related components, but if incorporated, the environmental considerations have a limited lens that overlook protecting natural environment resources from AMR pollution. Water especially has increasingly received more global attention, as it is recognized as a primary environmental vector for AMR spread and AMR-related diseases in low- and middle-income countries (LMICs) that have yet to achieve universal water, sanitation, and hygiene (WASH) access and wastewater treatment [35]. To address the environmental health planning gap in global approaches, country-level technical guidance has been developed to evaluate national One Health AMR progress [36] and the One Health Tripartite’s instruments and progress towards achieving GAP objectives [21,33,37].

AMR organisms are present ubiquitously and threaten all life on the planet, and thus environmental health is important to address in conjunction with AMR human and animal health stewardship efforts [32]. This study developed a novel AMR stewardship evaluation framework to address shortcomings in current AMR and water-related stewardship gaps. This framework evaluated 25 National Action Plans to determine thematic priorities in One Health AMR approaches and identified gaps nationally with regards to water-related stewardship to contribute to research and policy development for the environment dimension of the One Health framework. There is a need to expand AMR stewardship beyond its current purview to ensure that AMR prevention and mitigation efforts comprehensively address the diverse range of environmental reservoirs and dissemination pathways that promote AMR development. This review espouses integrating water management and four stewardship pillars (prevention, surveillance, mitigation, and innovation) to equitably protect diverse environmental (or ambient) waters, water supplies, and wastewater as one resource to protect public health.

## 2. Results and Discussion

### 2.1. NAP Evaluation Results

**Figure 1:**
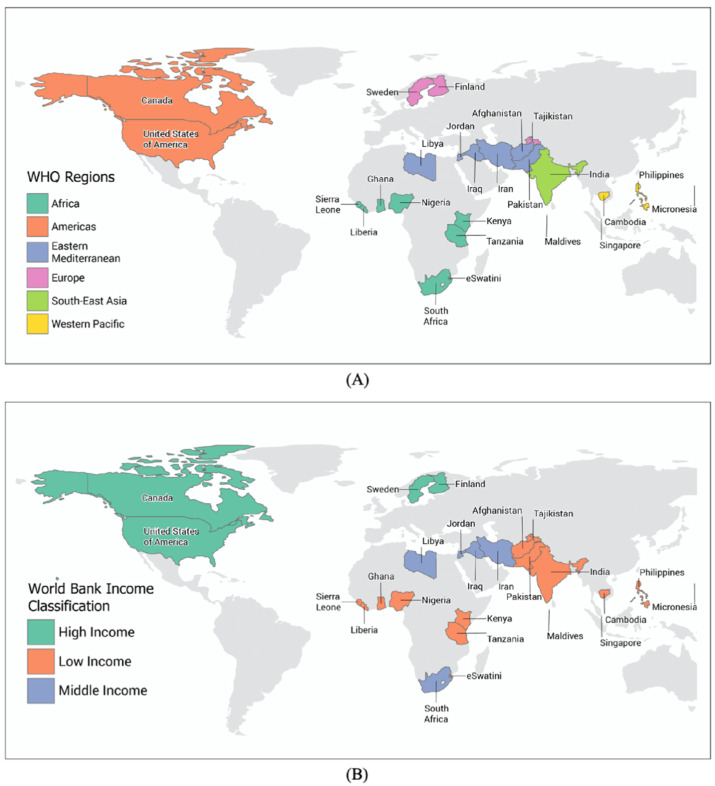
(**A**) Overview of the NAP case studies by global region. (**B**) Overview of the NAP case studies by income classification.

A selection of 25 NAPs were evaluated for AMR and water-related AMR stewardship to analyze global AMR thematic priorities and gaps using the One Health-One Water AMR stewardship framework developed in this study (Figure S1: One Health-One Water framework NAP evaluation results). NAPs were selected if (1) participated in the Tripartite AMR Country Self-Assessment Survey (TrACSS) (a global evaluative framework), (2) NAP is active at the time (2021) of the review, (3) available in English, (4) accessible online, and (5) included water-related terminology. The case studies represent all six WHO global regions [38]: Africa (AFR, 33%, *n* = 8), Americas (AMR, 8%, *n* = 2), Eastern Mediterranean (EMR, 24%, *n* = 6), Europe (EUR, 8%, *n* = 2), Southeast Asia (SEAR, 8%, *n* = 2), and Western Pacific (WPR, 16%, *n* = 4), and with regards to income level [39], 56% (*n* = 14) were LICs, 24% (*n* = 6) MICs, and 20% (*n* = 5) high-income countries (HICs) (Figure 1 and Table S1: NAP case study parameters). Results are reported by the following scorecard categories:

- One Health sectors (human, animal, and environment) and themes (human IPC, human antimicrobial consumption, use of antimicrobials in animals, food safety and security, use of antimicrobials in plants, environmental contamination, and clean water and sanitation);
- Water types (environmental (green), supply (blue), wastewater (brown));
- Pillars (mitigation, innovation, prevention, and surveillance).

The scorecards are divided into four quartiles with the first two lower quartiles indicating no to limited action and the upper two quartiles indicating outlined and implemented action, respectively.

### 2.2. One Health Sector: Human and Related Themes Findings

The human sector of One Health is concerned with monitoring the development and dissemination of AMR associated with human health as well as the impacts of AMR to human health. The human IPC and human consumption of antimicrobial themes fall within this sector, reflecting the focus of the Tripartite organizations for human-related AMR, specifically the WHO.

**Figure 2:**
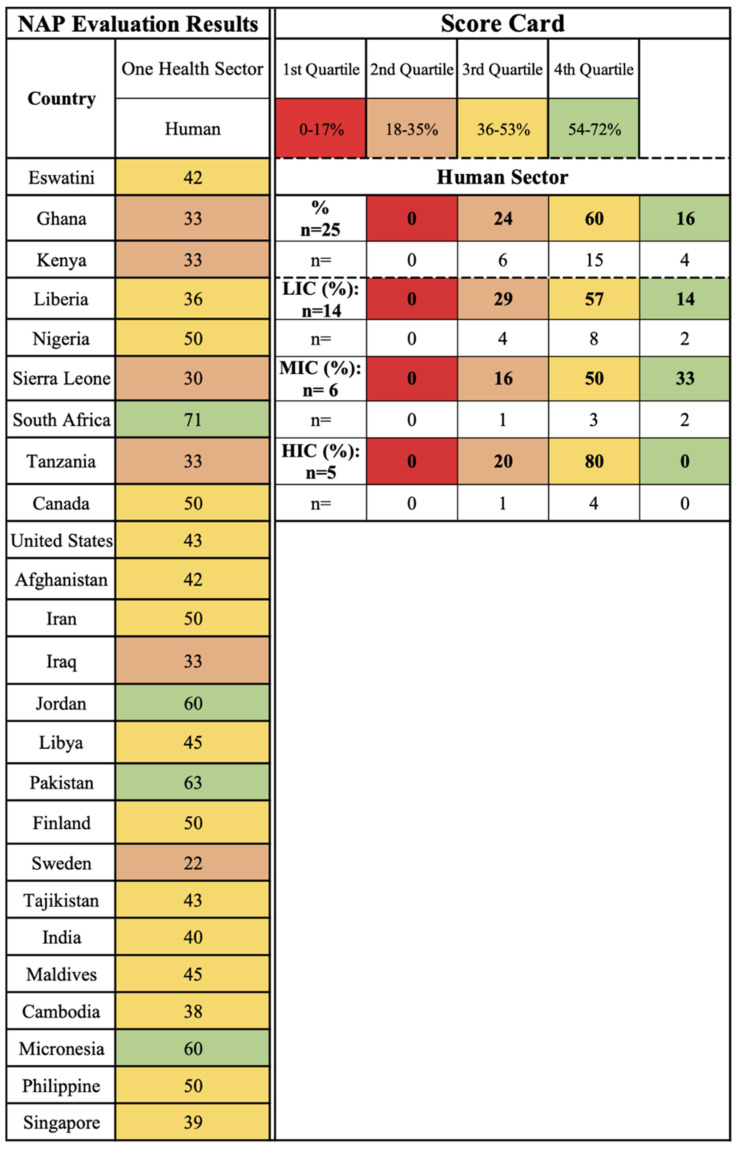
Human One Health sector scorecard by country and income level.

The human One Health sector is implemented into NAPs regardless of global region or country income status, with higher levels of implementation noted in the LICs (Pakistan and Micronesia) and MICs (South Africa and Jordan) case studies (Figure 2). HICs (80%, *n* = 4), MICs (50%, *n* = 3), and LICs (57%, *n* = 8) have the highest percentages of outlined activities within the third quartile, highlighting a universal focus on the human sector in NAPs.

**Figure 3:**
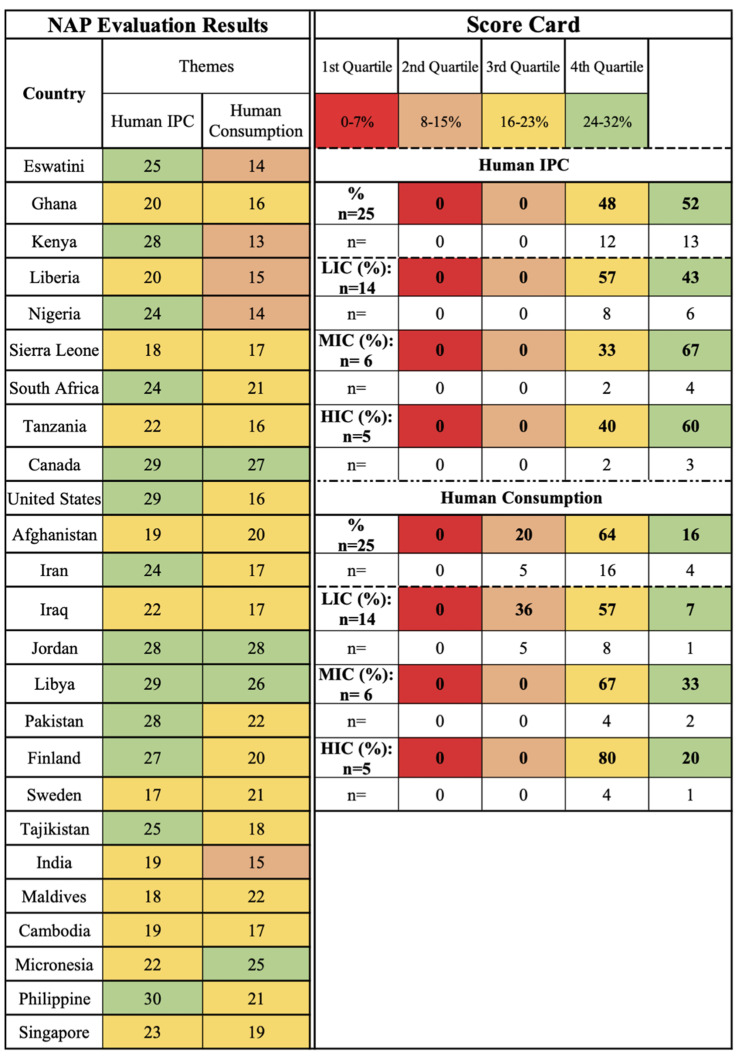
Human sector themes (human IPC and antimicrobial consumption) scorecard by country and income level.

The human IPC theme is primarily concerned with AMR in hospital settings and preventative measures to reduce the spread of human-related AMR. This theme has received the most attention with all case studies captured in the third (48%, *n* = 12) and fourth (52%, *n* = 13) quartiles of the scorecard (Figure 3). The majority of MICs (67%, *n* = 4) and HICs (60%, *n* = 3) are captured in the fourth quartile, and the majority of LICs (57%, *n* = 8) are captured in the third quartile followed by the fourth quartile (14%, *n* = 2). This suggests global prioritization of human IPC regardless of income level.

The human consumption of antimicrobials theme focuses on the availability of and the prescribing practices of antimicrobials for human health and is the third ranking theme with case studies captured in the third quartile (64%, *n* = 16), the second quartile (20%, *n* = 5), and the fourth quartile (16%, *n* = 4). The majority of LICs (57%, *n* = 8), MICs (67%, *n* = 4), and HICs (80%, *n* = 4) are captured in the third quartile, indicating outlined actions in NAPs, but there is slightly more focus in MICs and HICs than LICs (Figure 3). A 2021 report on the current state of antibiotic use highlights that LICs experience challenges in managing human antimicrobial consumption due to limited regulations, limited public education, and easy availability, which is reflected in these results [1]. The results of this study indicate the prioritization of human heath in relation to AMR as one of the primary foci of the NAPs in this review.

### 2.3. One Health Sector: Animal and Related Themes Findings

**Figure 4:**
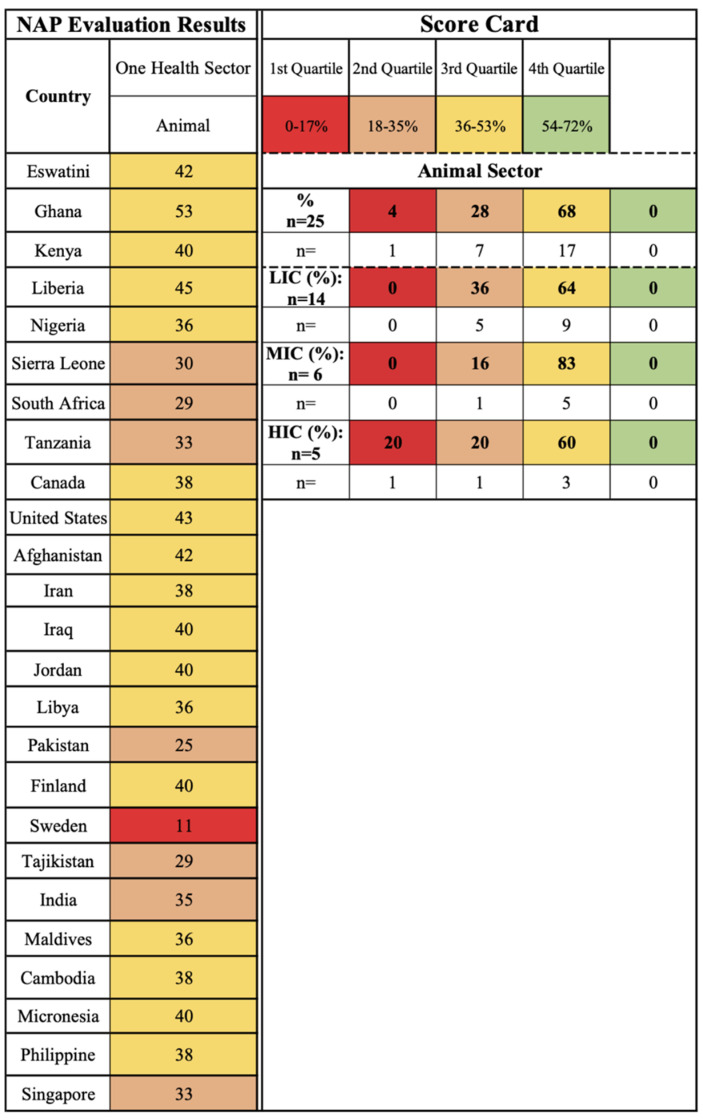
Animal One Health sector scorecard by country and income level. in plants) scorecard by country and income level.

The animal sector of One Health is concerned with monitoring the development of AMR in animals and this dissemination pathway impacting human health. Similar to the human sector, the majority of HICs (60%, *n* = 3), MICs (83%, *n* = 5), and LICs (64%, *n* = 9) are captured in the third quartile, indicating a universal focus on animal-related AMR within NAPs (Figure 4). The use of antimicrobials in animals, food safety and security, and use of antimicrobials in plants themes fall under this sector, reflecting the Tripartite interest in animal and food production, specifically through the FAO and OIE.

**Figure 5:**
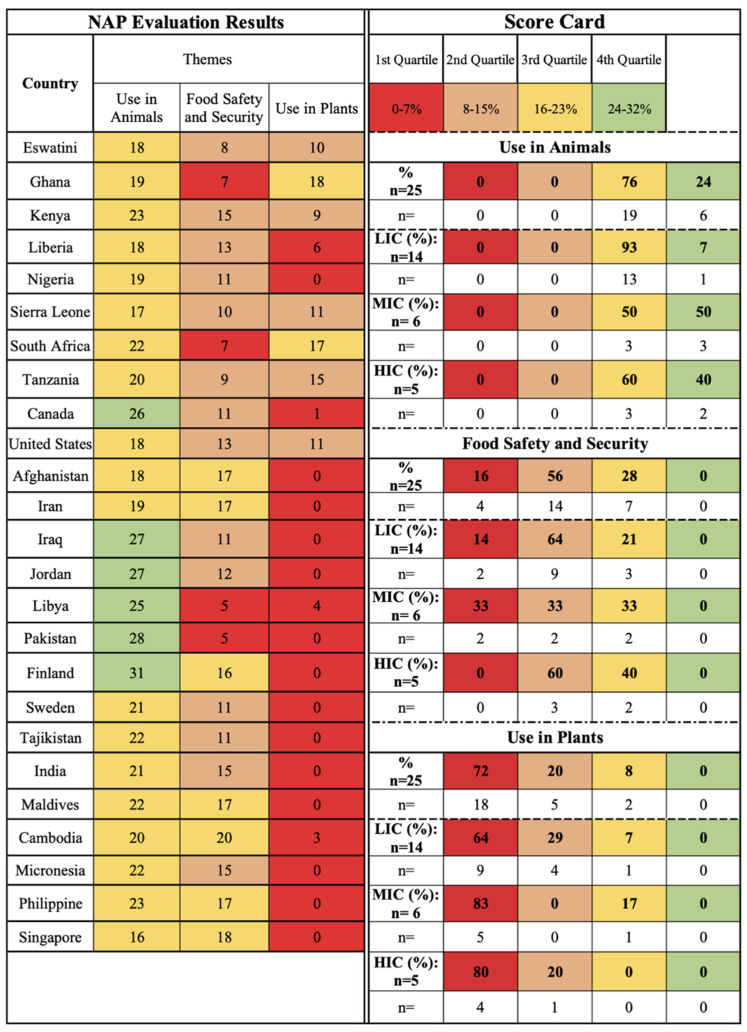
Animal sector themes (antimicrobial use in animals, food safety and security, antimicrobial use.

The use of antimicrobials in animals theme is focused on the availability and use of antimicrobials in animals, primarily food animal production, and is the second most prioritized theme after human IPC, with 76% (*n* = 19) of countries captured in the third quartile, and 24% (*n* = 6) in the fourth quartile. MICs (50%, *n* = 3) and HICs (40%, *n* = 2) are evenly captured in the top two quartiles, and the majority of LICs (93%, *n* = 13) are captured in the third quartile, indicating global attention on animal-related AMR risk (Figure 5). This theme in global guidance, as well as NAPs, is primarily concerned with livestock and food-producing animals, overlooking AMR risk linked to companion animals or wildlife. Regulations established by OIE are implemented in NAPs globally, prioritizing the management of this AMR source [40]. There is greater implementation in MICs and HICs, but this theme is highly prioritized regardless of income level.

The food safety and security theme is focused on residual antimicrobials from production in food products for human consumption. The majority of the case studies are captured in the second quartile (56%, *n* = 14), indicating limited attention for this theme globally (Figure 5). HICs, captured in the second (60%, *n* = 3) and third quartiles (40%, *n* = 2), have a greater focus than LMICs, captured in the first three quartiles; however, this theme is not prioritized in NAPs AMR stewardship.

Unlike its animal counterpart, the use of antimicrobials in plants theme, focused on their use for plant production and residuals that may be transported through consumption, is a thematic gap in NAPs, as it is primarily captured in the first quartile (72%, *n* = 18) for all case studies, indicating no or limited action (Figure 5). The 2020 Wellcome Trust AMR analysis also highlights that there is limited or no inclusion of this theme, reflected by limited to no available data to understand the scope of this dissemination pathway, but there is concern for human exposure through food products and contaminated water resources [23,41,42,43,44]. The Wellcome Trust analysis also notes that HICs regulate the use of antimicrobials in plant production, but MICs and LICs lack regulation and enforcement [23]. The results of this study, specifically the limited concern of this dissemination pathway in HICs (80%, *n* = 4, first quartile) as well as majority of MICs (83%, *n* = 5) and LICs (64%, *n* = 9) captured in the first quartile (Figure 5) continues to highlight this gap within One Health AMR stewardship.

The food safety and security and the use of antimicrobials in plants themes have received limited prioritization thus far, likely because the actions within these themes are not responses to reduce AMR but reduced exposure specifically through food production [23]. Per the Wellcome Trust analysis, supported by the results of this study, food products as well as plants, currently lack prioritization as AMR dissemination pathways outside of food animal production, limiting AMR stewardship [23]. Overall, the animal sector is a primary focus of One Health and is implemented across global regions and income levels with high evaluation scores in countries producing food animals and aquaculture, such as Ghana, the United States, Afghanistan, Finland, and Micronesia (Figure 4 and Figure 5).

### 2.4. One Health Sector: Environment, Related Themes Findings, and Water Types

**Figure 6:**
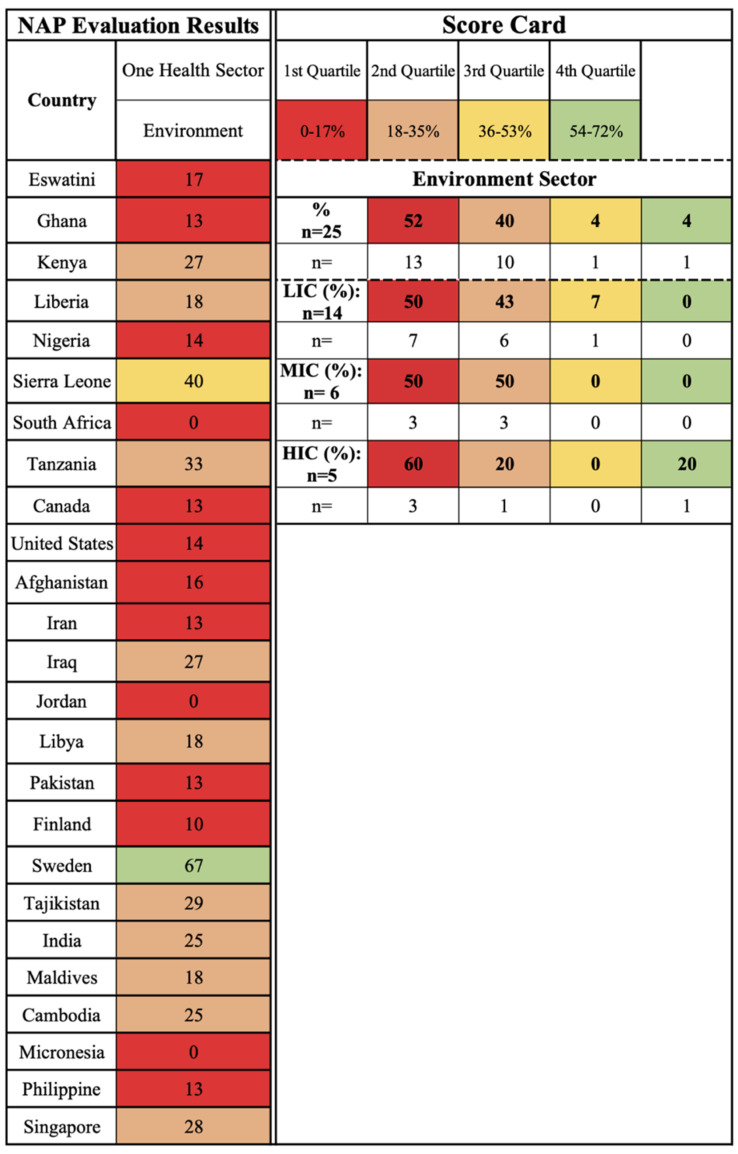
One Health environment sector scorecard by country and income level.

The environment sector of One Health is concerned with the environment acting as an AMR dissemination pathway for human exposure. The environment sector has received the least prioritization of the One Health sectors, as the majority of the case studies are captured in the first quartile (52%, *n* = 13), specifically HICs (60%, *n* = 3), MICs (50%, *n* = 3), and LICs (50%, *n* = 7), due to no or limited actions within their NAPs (Figure 6). The Tripartite organizations, aside from the United Nations Environment Programme, are not specifically focused on the environment, as reflected in the limited global guidance and inclusion in NAPs highlighted in this study. As a result, this sector and associated themes currently are poorly defined, particularly in relation to environmental reservoirs and dissemination pathways of AMR (water, soil, air) beyond wastewater management and WASH practices. The environmental contamination and clean water and sanitation themes fall under this sector.

**Figure 7:**
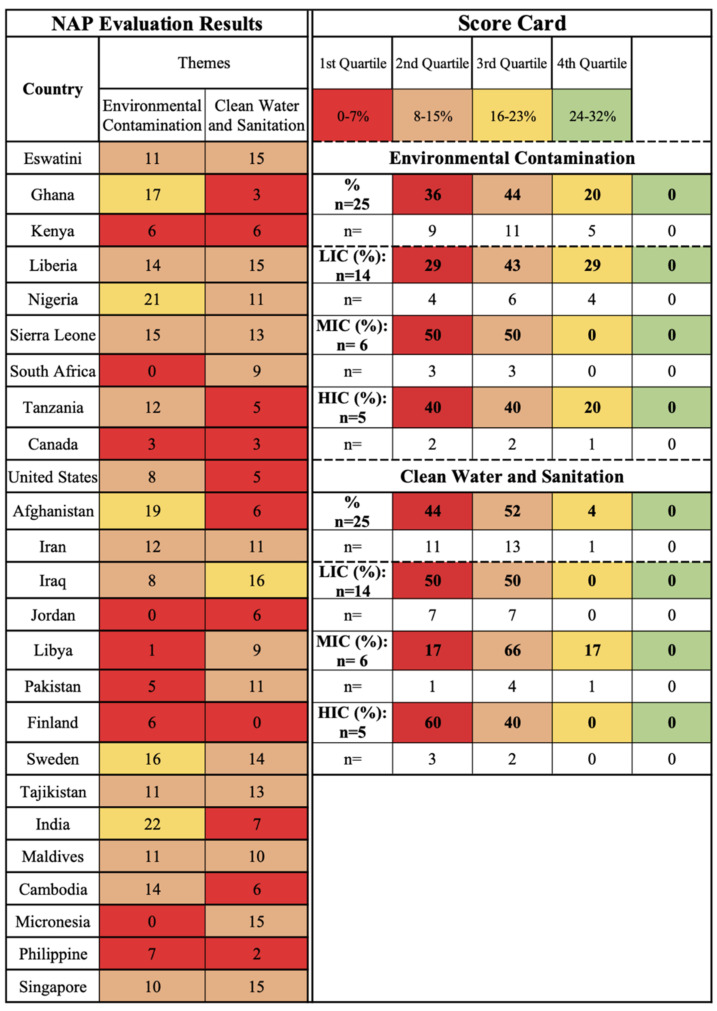
Environment sector themes (Environmental Contamination and Clean water and Sanitation) scorecard by country and income level.

**Figure 8:**
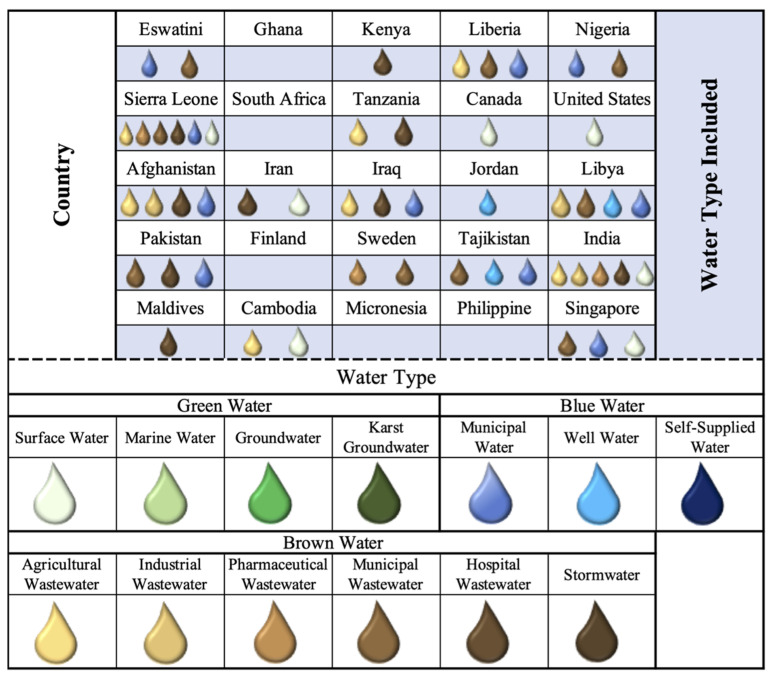
Breakdown of water categories and types included in the NAP case studies.

The environmental contamination theme is concerned with how environmental systems, including air, environmental waters, and soil contribute to AMR as a dissemination pathway, developmental area, and reservoir. The majority of the studies are captured in the first (36%, *n* = 9) and second (44%, *n* = 11) quartiles indicated that this theme is not prioritized in NAPs (Figure 7). With regards to water, pollution linked to wastewater is a primary consideration in the NAPs’ environmental contamination action planning. Wastewater contamination, in particular, is a focus, with 28% (*n* = 7) of NAPs highlighting agricultural wastewater considerations, 12% (*n* = 3) industrial, 12% (*n* = 3) pharmaceutical, and 36% (*n* = 9) municipal (Figure 8). Countries that have regulations and enforcement for environmental contamination are primarily HICs and MICs, including Sweden, Finland, and Iran, which focus on human and animal waste containment and wastewater treatment. India (LIC) is an exception due to the development of legislation for pharmaceutical wastewater in 2020, however, the 2020 Wellcome Trust report has highlighted implementation thus far is limited [23]. The contamination of environmental surface water (28% (*n* = 7) is a secondary consideration within this theme; however, marine water, groundwater, and karst groundwater environmental waters are overlooked excluding several sources of AMR from water-related AMR stewardship (Figure 8). There are currently no implemented AMR policies for environmental waters, including surface water, outlined in NAPs.

The clean water and sanitation theme is concerned with access to clean water supply to reduce AMR infections and the need for antimicrobial use primarily through WASH measures for humans and animals. The majority of the case studies are captured in the first (44%, *n* = 11) and second (52%, *n* = 13) quartiles, indicating no or limited action within NAPs regardless of the region or income level (Figure 7). LICs (Eswatini, Liberia, Nigeria, Sierra Leone, Pakistan, Tajikistan), MICs (Afghanistan, Iraq, Libya), and one HIC (Singapore) mention treating municipal water supply (40%, *n* = 10) as an AMR priority but had no outlined plans of action included in their NAPs (Figure 8). Currently, WASH measures focus primarily on healthcare facilities and communities, however, AMR is not prioritized in all WASH measures [23]. If this theme is included in NAPs, it is primarily through preventive measures for water supply implemented from a WASH perspective, in relation to IPC in hospital or community settings, as well as agricultural practices. The measures outlined within the NAPs include reducing AMR risk through handwashing practices, community education, training programs for healthcare workers, and the management of hospital wastewater (36%, *n* = 9; Figure 8); however, by the results of this study and in the screening process, which removed 17 NAPs from the sampling pool due to lack of water-related terms, this theme receives limited attention and action in AMR stewardship.

There is currently an overall lack of environmental and water-related AMR stewardship in NAPs. If environmental- and water-related actions are included within the NAPs, they focus on surveilling the transfer of AMR from anthropogenic activities, such as pharmaceutical production, wastewater treatment, and agricultural practices, through the environment to humans. Current AMR stewardship and research efforts are largely reactive measures to environmental AMR contamination, with limited proactive environmental protection measures, particularly for environmental waters, from becoming a reservoir and, in turn, dissemination pathway of AMR.

#### Environmental AMR Stewardship and Sustainable Development Goal 6 Relationships

**Figure 9:**
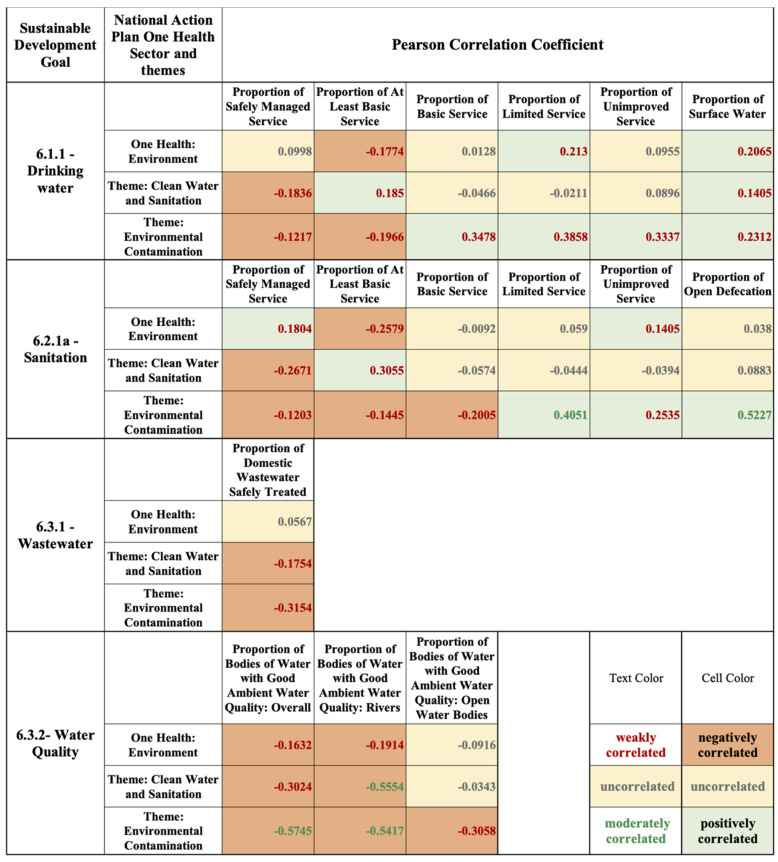
SDG 6 indicators and NAP case study evaluation scores (environment One Health sector, clean water and sanitation theme, environmental contamination theme) Pearson correlation analysis results.

The Pearson correlation coefficient (Figure 9) and linear regression (Figure S2: Linear regression graphs) were used to validate the framework evaluation findings for the One Health environment sector, clean water and sanitation theme, and environmental contamination theme scores for the NAP case study evaluations. These scores were analyzed with the Sustainable Development Goal (SDG) 6 indicators, of which SDG 6.1.1 (drinking water), 6.2.1a (sanitation), 6.3.1 (wastewater), and 6.3.2 (water quality) were considered. Currently, AMR is not integrated in the SDGs [45], however, SDG 6 speaks to broader water-related stewardship parameters, which reflect AMR stewardship priorities. To effectively integrate the environment and water resources into One Health, understanding the limitations and gaps of the current state of water-related AMR stewardship and water development progress is necessary and can guide future policy and the integration of water-related AMR stewardship.

The relationship between the selected NAP framework evaluation scores and SDG 6.1.1 indicates a mostly weak (0.1 ≥ |*p*| < 0.4) or uncorrelated (|*p*| < 0.1) relationship. The lower levels of treatment, including “basic service”, “limited service”, “unimproved”, and “surface water” show a weak positive correlation with the framework themes and One Health sector (Figure 9), primarily for LICs, where safe water supply is limited (Figure S2G–R). There are either weak or no relationships between the 6.2.1a SDG indicator and the NAP evaluation results (Figure 9). Similar to 6.1.1, the high levels of treatment are represented primarily by HICs, and the lower levels of treatment are represented by MICs and LICs (Figure S2S–AJ). The weak correlation (*p* = 0.3055) between “at least basic service” and the clean water and sanitation theme reveals that MICs and LICs have stronger, albeit limited, plans for clean water and sanitation AMR stewardship than HICs (Figure 9; Figure S2W). In addition, for this indicator, the environmental contamination theme is moderately correlated (0.4 ≥ |*p*| < 0.6) with “limited service” (*p* = 0.4051) (Figure 9; Figure S2AD), weakly correlated with “unimproved service” (*p* = 0.2535), and moderately correlated with “open defecation” (*p* = 0.5227) (Figure 9; Figure S2AJ). The 6.3.1 dataset includes the proportion of domestic wastewater flow safely treated, overlooking industrial, pharmaceutical, hospital, agricultural, and stormwater. There is no correlation with the One Health environment sector and a negative correlation with the clean water and sanitation theme, as well as the environmental contamination theme (Figure 9; Figure S2AK–AM). This indicator has limited data available, with only 16 of the case studies reporting data, primarily MICs and HICs. There are either no or weak correlations between the SDG 6.3.2 indicator and the NAP evaluation results (Figure 9; Figure S2AN–AV). This indictor also has limited data with only 13 of the case studies reporting data.

The correlation analysis and linear regression results of this study support the findings of the NAP evaluations by highlighting a thematic prioritization alignment between the SDG 6.1.1, 6.2.1a, and 6.3.1 indicators and the NAPs. The positive correlations with the limited water development progress outlined in the 6.1.1 and 6.2.1a indicators (drinking water and sanitation) and the NAP evaluations, particularly in LMIC, highlights the prioritization of AMR stewardship in LMICs, particularly for water supply. The countries with less water development progress prioritize AMR stewardship needs, however, stewardship action is limited, as highlighted in the NAP evaluations. There is a need for better stewardship of AMR in LMICs, which translates to prioritizing water development progress for these indicators, and, in turn, reducing AMR exposure pathways. Wastewater (indicator 6.3.2) is highlighted as an AMR dissemination pathway in NAPs. Implementation actions for wastewater are prioritized in LMIC due to limited treatment technology, which is reflected by the limited SDG indicator data available for LMIC. Wastewater is not prioritized in HIC due to available treatment technology, which is reflected by the available indicator data and lack of correlation. Similar to the prioritization of human and animal health in current AMR stewardship, environmental waters (6.3.2), as indicated by negative correlations, receive limited attention for AMR stewardship and water development progress. The findings of this analysis support the NAP evaluation results, highlighting that the environment and water resources are overlooked beyond clean WASH. There is a greater prioritization of water-related AMR stewardship in LMIC than HIC, due to extensive exposure pathways, however, there is limited action towards these measures, inhibiting a successful One Health approach.

### 2.5. Stewardship Pillar-Related Findings

**Figure 10:**
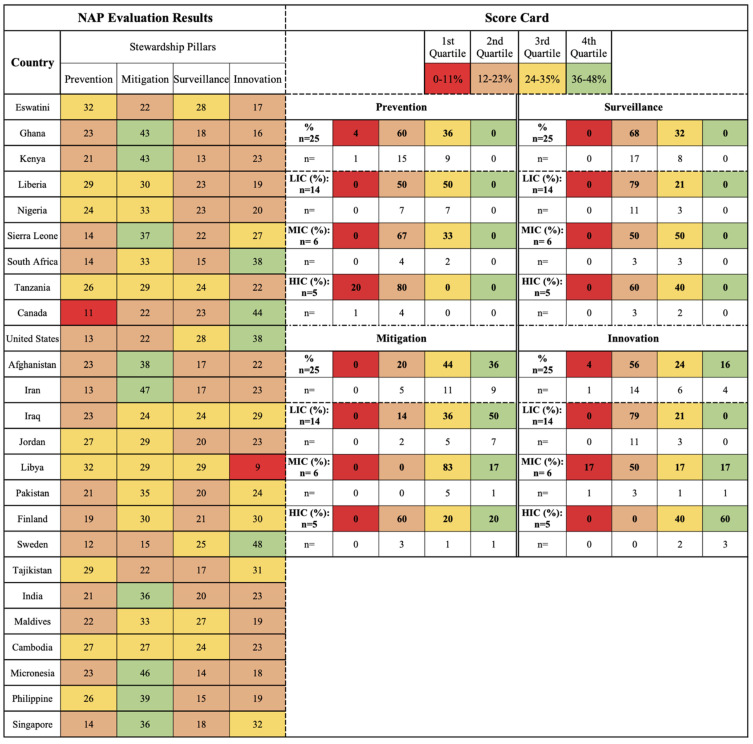
Stewardship pillars (mitigation, innovation, prevention, surveillance) scorecard by country and income level.

The four stewardship pillars outlined in this framework are integrated in the case studies (Figure 10). Mitigation activities are centered around regulations and management. Majority of LICs (50%, *n* = 7) are captured in the fourth quartile for mitigation due to developed and implemented AMR polices and regulations primarily for human IPC and antimicrobial consumption, as well as animal antimicrobial consumption. Mitigation activities are also prioritized in MICs (83%, *n* = 5), which are captured in the third quartile. The greater implementation of this pillar in LICs and MICs than in HICs reflects a prioritization of human health due to lower levels of sanitation in health care facilities and animal production and, in turn, greater risk of infection.

The innovation pillar evaluates NAPs for research progress, AMR understanding, and data sharing networks. A greater prioritization for innovation is noted in the HIC case studies (60%, *n* = 3) captured in the fourth quartile (Figure 10). The majority of MICs are captured in the first (17%, *n* = 1) and second (50%, *n* = 3) quartiles, and LICs are primarily captured in the second quartile (79%, *n* = 11). The overall prioritization and feasibility of innovation is bias towards HICs and MICs in the Americas and European WHO regions. The prevention pillar is centered around proactive actions, including public outreach and education, as well as professional training programs. LICs are equally captured in the second and third quartiles (50%, *n* = 7), MICs are primarily captured in the second quartile (67%, *n* = 4) followed by the third quartile (33%, *n* = 2), and HICs are primarily captured in the second quartile (80%, *n* = 4) (Figure 10). The surveillance pillar is centered around monitoring AMR within One Health and utilizing data to influence actions within the other pillars. HICs and MICs are evenly captured in the second (60%, *n* = 3; 50%, *n* = 3, respectively) and third quartiles (40%, *n* = 2; 50%, *n* = 3, respectively), whereas LICs are primarily captured in the second quartile (79%, *n* = 11) (Figure 10). The development and implementation of surveillance instruments is targeted in all NAP case studies, but LICs are still establishing plans for these systems.

The implementation of the stewardship pillars aligns with the thematic priorities of AMR stewardship, as the stewardship pillars are currently focused on human and animal health actions, continuing to overlook the environment. In addition, the resource demanding pillars (innovation and surveillance) are prioritized in HICs and MICs and the less resource demanding pillars (mitigation and prevention) are prioritized in LMICs. The inclusion of all four pillars is vital for successful water stewardship and the snapshot provided though this evaluation indicates progress is still needed for all sectors of One Health, particularly the environment and water resources.

### 2.6. Realizing an AMR One Health Approach

The developed AMR stewardship framework is a useful tool for identifying priorities and gaps in current global and national AMR guidance. This review concurs with prior global assessments that the human and animal health sectors and themes in NAPs are prioritized over the environmental component of One Health (environmental contamination, and clean water and sanitation) and the use of antimicrobials in plants. The environment sector of One Health is complex and encompasses a variety of sub-environments (soil, air, water) that are not equitably accounted for in NAPs, and by extension, global guidance. This review highlights especially the significance of addressing water as an environmental AMR driver given its critical role in supporting environmental, human, and economic systems. To successfully include advance One Health AMR approaches, national plans and global guidance need to go beyond current human and animal sector prioritization and give greater attention and resources to interconnected environmental AMR drivers that threaten to setback human development.

In order to holistically integrate environmental health into One Health, environmental stewardship guidance needs to be developed, incorporated, and supported at the global level. The application of this framework can aid policymakers and multilateral agencies in developing more holistic AMR stewardship plans integrating the gaps highlighted in current plans. In hindsight, it is not surprising that AMR stewardship thus far largely focuses on human and animal health, as the Tripartite organizations leading global and guiding national efforts are focused on these sectors. As of 2018, the Tripartite has expanded to include the United Nations Environment Programme (UNEP) within the Tripartite Plus to better incorporate the environment and increase national level action [46]. However, the findings in this study highlight limited environmental focus at the national level. This suggests that while the significance of the environment sector in the dissemination of AMR has been acknowledged globally, there is still a huge gap in planning and action to achieve a One Health AMR approach [23,32,34,47].

Reflected by the findings of this study, thus far, the role of the UNEP in the Tripartite Plus has been limited in available global and national guidance. To better address the environmental sector, the UNEP needs to actively integrate environmental AMR guidelines and actions, specifically for water resources, into global guidance as well as aid in integration into national action plans. Along with, enacting the stewardship pillars into environmental AMR actions, though public awareness, surveillance programs, regulations, and development of removal technologies is necessary for successfully implementing the environment sector.

### 2.7. One Water AMR Stewardship

More holistic and integrated water management considerations are needed in AMR stewardship plans to especially benefit LMICs that are at greater risk of water-related AMR threats. The management of water supply and wastewater receives the most attention within LMIC NAPs given the threat of limited treatment of drinking water and wastewater from various sources contaminating water supplies [21] that could otherwise limit AMR transmission and reduce the need for antimicrobial use [23]. Global policy guidance highlights and provides limited guidelines for protecting the environment—in particular, hospital and community-based water, sanitation, and hygiene (WASH) interventions and wastewater treatment in combating AMR, however, this guidance overlooks the role of environmental waters as reservoirs and dissemination pathways [21]. The NAP evaluations and SDG 6 validation highlights a severe gap within AMR stewardship and prevention with regards to environmental waters, despite public health experts from the Wellcome Trust acknowledging water systems as a hotspot for AMR development, environmental dissemination, and human transmission [23]. Oversight of this dissemination pathway is especially concerning for LMICs, as they tend to have limited availability of clean water and limited water treatment at scale, making the population health threat of AMR in environmental waters even greater [23].

Current water-related AMR guidance reflects siloed water management, with water sources, supplies, or waste managed individually. Water, however, should be managed in an integrated way throughout the water cycle—from the environment through use and discharge because the contaminants, such as AMR, are also transported throughout the cycle, impacting human and environmental health. Current water- related guidance highlights the potential impact of AMR in water supply and wastewater but provides limited guidelines and actions to manage AMR. As a result, the broad-based statements and regulations pertaining to water resources are not conducive to effective water resource management and protection because water resources are impacted by different anthropogenic practices, receive different levels of treatment, and are sourced and utilized by location specific needs [47,48].

The proposed One Health-One Water AMR stewardship framework accounts for water’s integrated management throughout the water cycle. One Water is premised on the interconnections of different phases of the water cycle (environmental waters, water supply, and wastewater), with all types of water equitably treated as being a potential AMR reservoir and dissemination pathway. The framework dually evaluates the primary focuses and shortcomings of AMR and water-related AMR stewardship to aid policymakers and multilateral agencies in further developing holistic stewardship for human, animal, and environmental health. Highlighting these strengths and weaknesses also emphasizes areas for improvement in current AMR stewardship for water resources, particularly environmental waters.

## 3. Methods

### 3.1. One Health-One Water AMR Stewardship Framework: Integrating a Water Stewardship Lens

A literature review of existing global AMR instruments developed by the United Nations (including the tripartite organizations) and global non-governmental agencies [23,25,28,33,36,37,45,49,50] was conducted to understand the current state of One Health and AMR stewardship. This study primarily builds upon two One Health and AMR stewardship frameworks from the 2020 Wellcome Trust [23] and the IACG’s AMR Framework for Action [45] in relation to the reviewed global instruments. Both frameworks outline the focus areas for AMR stewardship with respect to the Global Action Plan (GAP) objectives and provide detailed goals and enablers to achieve the objectives. The Wellcome framework outlines the modified specific themes for:

- human infection prevention and control (IPC);
- clean water and sanitation;
- food safety and security;
- environmental contamination;
- human consumption of antimicrobials;
- use of antimicrobials in animals;
- use of antimicrobials in plants.

The Wellcome Trust framework also outlines enablers adapted from the IACG framework levers, which are necessary to achieve thematic actions:

- surveillance;
- innovation (discovery and translation research, diagnostics, therapeutics, vaccines, medicine quality, clinical trial networks);
- national action;
- global governance.

The IACG framework’s levers (description of how the content areas/themes can be addressed) for each of the themes includes:

- awareness and capability building;
- surveillance;
- funding and financial incentive;
- policy and regulation;
- championing and piloting.

While comprehensively thematic, the Wellcome Trust and IACG frameworks are primarily utilized for human and animal health guidance, providing limited guidance for including the environment sector or water resources in holistic AMR stewardship beyond WASH measures [23,31,45]. This translates to global AMR stewardship being primarily developed to address IPC as well as human and animal antimicrobial use concerns, which overlooks the environmental health component of One Health. AMR-related global and national instruments developed by the Tripartite have acknowledged the need for actively incorporating the environment sector, particularly water resources, into these instruments as a dissemination pathway of AMR outside of the human and animal sectors, but guidance remains largely broadly brushed statements with AMR action planning guidance focused on WASH provisions to protect human health [25,31,33,36,37,45,51]. This overlooks the need for specification of recommendations for managing and protecting the wide range of environmental and water risks outside of a human IPC and animal context.

**Figure 11:**
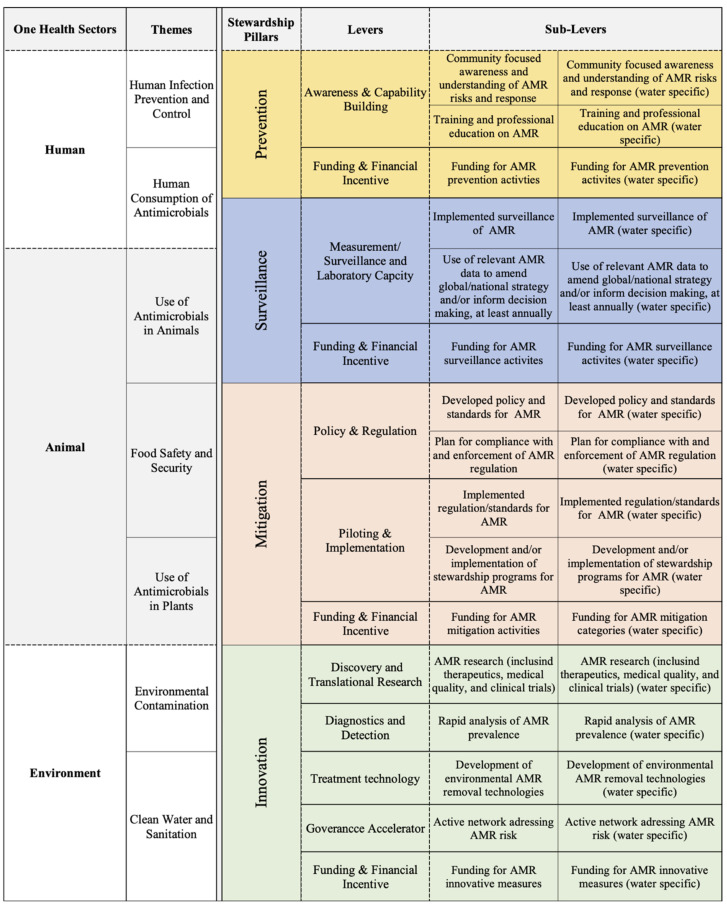
The One Health-One Water AMR stewardship framework.

To address limitations of these AMR frameworks, a One Health-One Water AMR stewardship framework and evaluation tool that comprehensively and equitably covers human, animal and environmental aspects of AMR risk was developed, incorporating aspects of the Wellcome Trust and IACG frameworks (Figure 11). The framework is intended to be utilized as an evaluation tool to determine strengths and weakness of current AMR stewardship. Highlighting these strengths and weakness can aid in developing effective AMR stewardship, particularly for the protection and management of water resources and public health.

The framework integrated each of the One Health Sectors—human, animal, and environment—as umbrella categories for the themes as well as to determine the primary focus areas of One Health in current AMR stewardship. The themes defined in the IACG and the Wellcome Trust frameworks were integrated into the One Health-One Water framework. The enabler theme from the Wellcome Trust framework was adapted into four stewardship pillars—prevention, surveillance, mitigation, and innovation—which are defined as key components of stewardship, particularly for water management and protection. The prevention pillars encompass stewardship activities intended to prevent AMR contamination, the surveillance pillar encompasses monitoring activities, the mitigation pillar encompasses activities focused on reactive management measures, and the innovation pillar encompasses reactive and proactive measures. The levers outlined in the IACG framework were integrated into the One Health-One Water framework within the stewardship pillar categories. To understand the funding aspect of stewardship, a funding and financial incentive lever was added to each pillar for this study’s framework (see Figure 11).

To effectively assess the progress of the established One Health sectors, themes, and pillars, sub-levers were developed to provide an evaluation component to the framework [23,45]. The sub-levers—created from aspects of the Wellcome Trust framework’s enabler theme and global instruments [23,36,45]—are categorized within the lever categories and are utilized as the evaluation criterion for this framework (see Figure 11). A water-related sub-lever compliments each appropriate sub-lever to evaluated water-related AMR stewardship alongside AMR stewardship.

To enhance water-related intervention planning with respect to AMR and stewardship, water definitions were developed within this study for different water types that are known to be AMR dissemination pathways or reservoirs [7,11,12,15,24,52]. The one water concept considers water resources throughout the water cycle, as well as contaminants introduced and transported with water resources. Specifying water types is necessary to effectively target policies, as well as regulate and protect water resources [53] in an integrated manner, from source to treatment [54]. The study’s 13 water types represent water from the 3 main roles water plays within society and nature: resource, supply, and contaminant:

- The Water Resource category indicates any of the ambient or environmental waters (surface, marine, groundwater, karst groundwater) which can be utilized for human, industrial, agricultural, or ecosystem use. Environmental waters refer to the diverse bodies of water present in human environments (natural or man-made), which can support ecosystem biodiversity and services, be utilized as a water supply, and/or receive wastewater discharge. Water resources are from open systems and are not treated but have the potential to be utilized.
- The Water Supply category indicates any water that has been collected from a water resource and is intended for human/industrial use or consumption.
- The Water Contaminant category indicates water that has been used and is not intended as a resource or supply but contributes to both types with and without treatment.

**Figure 12:**
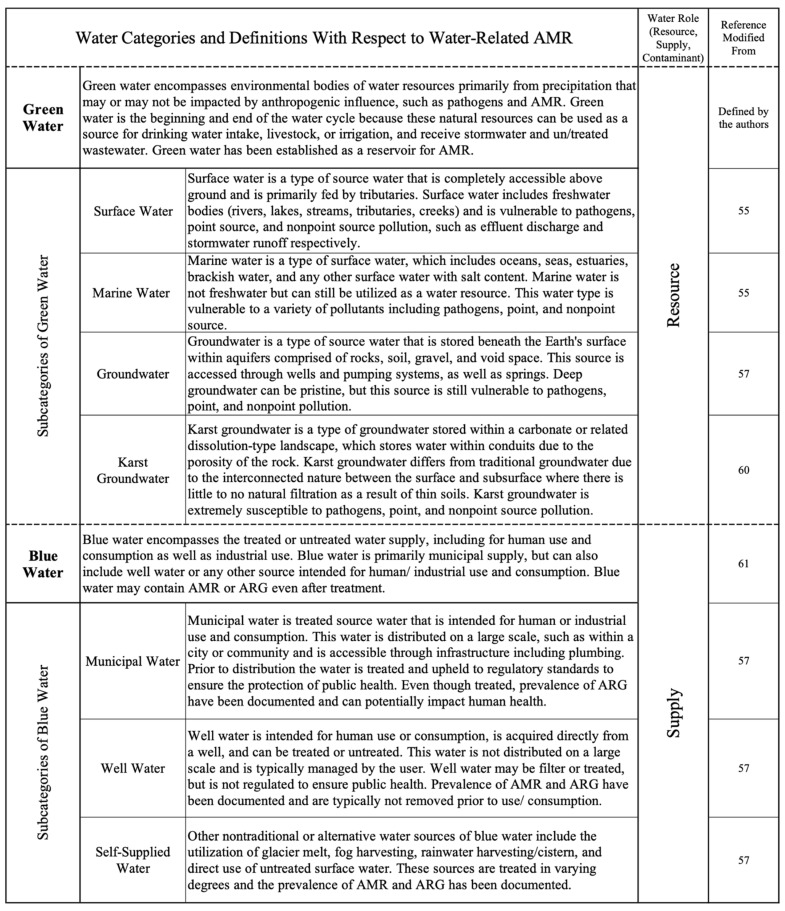

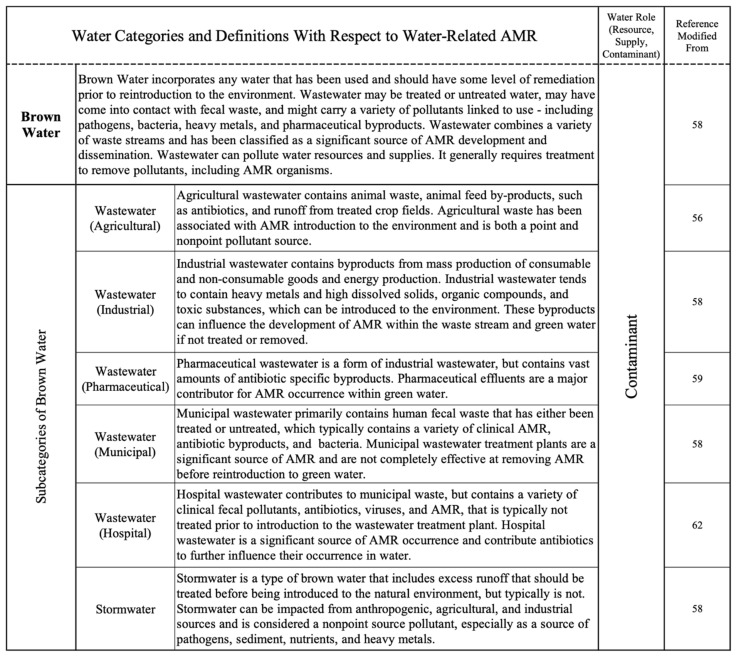
Defined water categories and types associated with water-related AMR.

Where possible, water type definitions (see Figure 12) were adapting from preexisting definitions [55,56,57,58,59,60,61,62]. While the categories and types are targeted towards AMR policy and management, these classifications can be universally applied in any water-related policy.

### 3.2. National Action Plan Case Study Selection

National Action Plans were targeted for this study in order to determine general One Health and thematic prioritization, as well as to determine, if any, water-related AMR stewardship gaps. The NAPs utilized in this study were accessed from the WHO Library of AMR National Action Plans. The case studies were selected if the following criteria were met:

- The country participated in the Monitoring Global Progress on Antimicrobial resistance: 2019–2020 Tripartite AMR Country Self-Assessment Survey (TrACSS), a comprehensive list of actively involved countries;
- The NAP was fully developed and currently implemented. If multiple versions available, only the currently implemented NAP was considered;
- The NAP was published in English;
- The NAP was accessible/available online;
- The NAP included the terms “water”, “sanitation”, or “hygiene” outside of the background/introduction statement.

**Figure 13:**
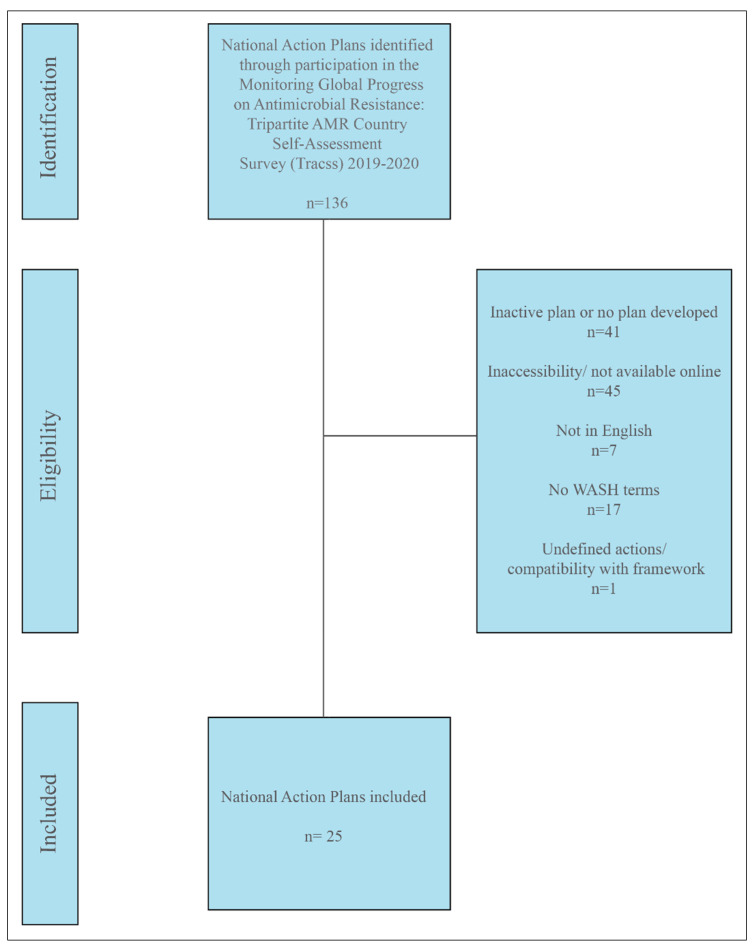
Prisma diagram reflecting the NAP case study screening process.

Ultimately, 25 NAP case studies were evaluated to understand the focus of these current plans concerning AMR and water-related AMR stewardship (Figure 13).

### 3.3. Data Analysis

#### 3.3.1. Framework Evaluation and National Action Plan Scorecard

**Figure 14:**
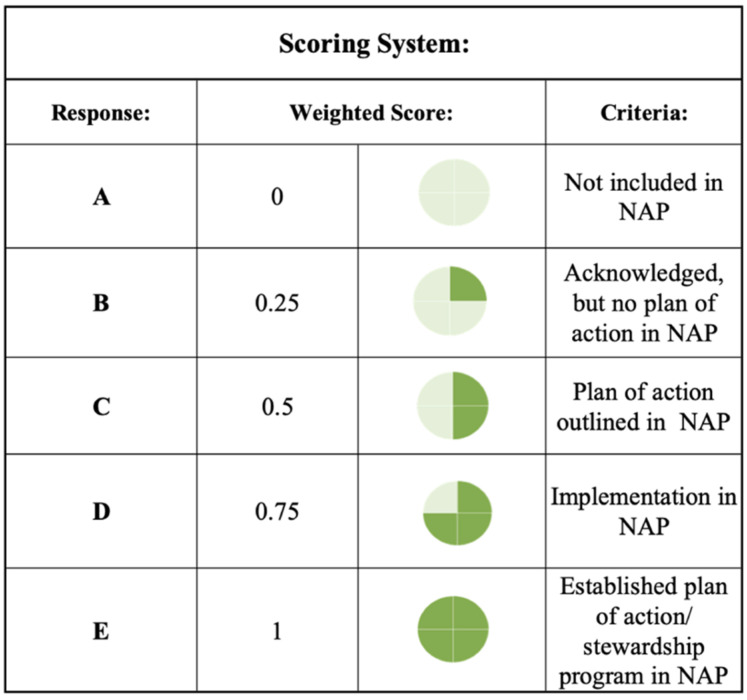
One Health-One Water AMR stewardship framework scoring criteria.

To evaluate the attention towards AMR and water-related AMR stewardship an evaluation of the NAP case studies was conducted using the study’s One Health-One Water AMR stewardship framework. Each of the included NAPs was scored per One Health sector in relation to the themes and pillars in relation to each of the sub-lever categories based on the implementation status of thematic activities (Figure 14). A total score was then calculated per NAP for each of the One Health sectors, pillars, and themes. Scorecards for the One Health sectors, pillars, and themes were developed (Figure 2, Figure 3, Figure 4, Figure 5, Figure 6, Figure 7 and Figure 10), which were divided into four quadrants in relation to the evaluation scores, to compare AMR stewardship progress between the case studies. To further understand the progress of AMR stewardship at the country income classification level, percentages of each classification (LIC, MIC, and HIC) were standardized and compared in relation to each of the sectors, pillars, and themes. The scores highlight the overall focus of AMR and any water-related AMR stewardship for each NAP. Guidelines were developed for the framework to aid in theme parameters and framework scoring as well as systematic comparison between NAPs (Table S2: Framework guidelines, Table S3: Framework scoring guidelines).

#### 3.3.2. Framework Validation with Sustainable Development Goal Correlation

The framework evaluation findings for the human and animal sectors and themes are validated through triangulation with preexisting literature. The environment sector and related themes are supported by literature as well, however, the limited focus on this sector required further validation. The sustainable development goal (SDG) 6, an indicator for water development progress, and the thematic actions of environmental AMR stewardship highlighted in this study were correlated to validate the environmental and water-related aspect of the Framework. SDG 6 indicators were selected because the UN Interagency Coordination Group (IACG) AMR framework for action has highlighted the parallel between AMR stewardship and water development progress [31,45]. This method was also selected as previous studies have analyzed SDG indicator data in comparison to other factors using correlation analysis [63,64,65]. A Pearson correlation analysis [66] was used to determine correlations between the framework evaluation scores for the environment One Health sector, the clean water and sanitation theme, and the environmental contamination theme with indicators of SDG 6. Indicator data were collected for 6.1.1 Drinking Water [67], 6.2.1a Sanitation [68], 6.3.1 Wastewater [69], and 6.3.2 Water Quality (excluding groundwater due to data only available for five of the case studies) [70] for each of the case study countries if available. The SDG data were provided by the Joint Monitoring Programme for Water Supply, Sanitation and Hygiene, WHO, United Nations Habitat, and the United Nations Environment Programme (Table S4: Sustainable development goal data). Linear regression [71] was also implemented for these data with the R squared value to measure the goodness of fit (also referred to as the coefficient of determination). The correlations, linear regressions, and R squared values were implemented in Python 3 using Numpy. The code was accessed on 30 October 2021 and can be found at https://github.com/vincihb/nap_correlations.

### 3.4. Limitations

A limitation of this study is the selection of case studies from the TrACSS, which does not include all NAPs, but only participating countries. In addition, the WASH terminology inclusion criterion might show a greater inclusion of the environment as well as water resources than reflected globally. The NAP may have been vague and not included or fully outlined thematic activities, such as policies or surveillance systems, skewing the evaluation results. The themes within the human (human IPC and human consumption of antimicrobial) and animal (antimicrobial use in animals, food safety and security, antimicrobial use in plants) One Health sectors were not always distinguishable within NAP activities due to broad statements within the NAP. In order to distinguish between the themes, thematic language and responsible entities, if included, were utilized.

## 4. Conclusions

This research is a snapshot of the current state of AMR thematic priorities and AMR water-related stewardship in global policy guidance and National Action Plans. The human and animal sectors receive the most attention while the environment sector, food safety and security, and antimicrobial plant use have limited focus in current guidance and policy. The integration of these latter themes into future guidance and action plans is necessary to manage potential environmental and foodborne AMR dissemination pathways.

With regards to water, the One Health-One Water AMR stewardship framework was a useful tool to evaluate AMR priorities in NAPs and gaps in AMR and water-related stewardship. This review found that environmental waters are overlooked in AMR stewardship and national water development plans that focus on WASH access and wastewater treatment. This oversight poses a major public health threat in LMICs, where half a billion people rely on environmental waters that potentially are heavily polluted from domestic, industrial, and agricultural discharge to meet their daily water needs. Utilizing the study’s framework can help policymakers and multilateral agencies broaden the water AMR lens beyond WASH and human IPC measures, so that environmental waters, water supplies and wastewaters are each accounted for as both dissemination pathways and reservoirs.

The discussion on AMR is constantly evolving, especially as the role of the environment and water resources is better understood in terms of AMR development, dissemination, and transmission. The integration of water resources and One Health for AMR stewardship is vital for the protection of human and environmental health through safe and secure water resource management and protection. AMR is a global problem that requires action at the global and at the national level in both LMICs and HICs alike [72]. Water-related AMR can be managed and support One Health objectives through the holistic integration of One Health, water resources and the four stewardship pillars.

## Data Availability

The data presented in this study are available in [Figures S1 and S2, Table S4] and at https://github.com/vincihb/nap_correlations, which was accessed on 30 October 2021.

## Supplementary Materials

The following are available online at https://www.mdpi.com/article/10.3390/antibiotics11010063/s1, Figure S1: One Health-One Water framework NAP evaluation results, Figure S2: Linear regression graphs, Table S1: NAP case study parameters, Table S2: Framework guidelines, Table S3: Framework scoring guidelines, Table S4: Sustainable development goal data.
