# Supplementary material for: Antimicrobial Resistance and Environmental Health: A Water Stewardship Framework for Global and National Action
Source: Antibiotics (Basel). 2022 Jan 5;11(1):63. doi: 10.3390/antibiotics11010063 (PMC8773023; doi:10.3390/antibiotics11010063)
Supplement: Supplementary file 1 [file antibiotics-11-00063-s001.zip › Figure S1_ONeHEalth-OneWater-AMR_StewardshipFrameworkEvaluationResults.pdf]

Country	WHO Region: World Bank Country Income Classification	NAP Timeframe	NAP Version	Water Type Included	AMR One Health Sector			Pillars				Themes						
												Reduce Need and Unintentional Exposure				Optimize Use of Medicines		
					Human	Animal	Environment	Prevention	Mitigation	Surveillance	Innovation	Human Infection Prevention and Control	Clean Water and Sanitation	Food Safety and Security	Environmental Contamination	Human Consumption of Antimicrobials	Use of Antimicrobials in Animals	Use of Antimicrobials in Plants
Eswatini	AFR: LIC	2018-22	1		42	42	17	32	22	28	17	25	15	8	11	14	18	10
Ghana	AFR: LIC	2017-21	2		33	53	13	23	43	18	16	20	3	7	17	16	19	18
Kenya	AFR: LIC	2017-22	1		33	40	27	21	43	13	23	28	6	15	6	13	23	9
Liberia	AFR: LIC	2018-22	1		36	45	18	29	30	23	19	20	15	13	14	15	18	6
Nigeria	AFR: LIC	2017-22	1		50	36	14	24	33	23	20	24	11	11	21	14	19	0
Sierra Leone	AFR: LIC	2018-22	1		30	30	40	14	37	22	27	18	13	10	15	17	17	11
South Africa	AFR: MIC	2018-24	2		71	29	0	14	33	15	38	24	9	7	0	21	22	17
Tanzania	AFR: LIC	2017-22	1		33	33	33	26	29	24	22	22	5	9	12	16	20	15
Canada	AMR: HIC	2017-	1		50	38	13	11	22	23	44	29	3	11	3	27	26	1
United States	AMR: HIC	2020-25	2		43	43	14	13	22	28	38	29	5	13	8	16	18	11
Afghanistan	EMR: LIC	2017-21	1		42	42	16	23	38	17	22	19	6	17	19	20	18	0
Iran	EMR: MIC	2016-21	1		50	38	13	13	47	17	23	24	11	17	12	17	19	0
Iraq	EMR: MIC	2018-22	1		33	40	27	23	24	24	29	22	16	11	8	17	27	0
Jordan	EMR: MIC	2018-22	1		60	40	0	27	29	20	23	28	6	12	0	28	27	0
Libya	EMR: MIC	2019-23	1		45	36	18	32	29	29	9	29	9	5	1	26	25	4
Pakistan	EMR: LIC	2017-	1		63	25	13	21	35	20	24	28	11	5	5	22	28	0
Finland	EUR: HIC	2017-21	1		50	40	10	19	30	21	30	27	0	16	6	20	31	0
Sweden	EUR: HIC	2020-23	2		22	11	67	12	15	25	48	17	14	11	16	21	21	0
Tajikistan	EUR: LIC	2018-	1		43	29	29	29	22	17	31	25	13	11	11	18	22	0
India	SEAR: LIC	2017-21	2*		40	35	25	21	36	20	23	19	7	15	22	15	21	0
Maldives	SEAR: MIC	2017-22	1		45	36	18	22	33	27	19	18	10	17	11	22	22	0
Cambodia	WPR: LIC	2019-23	2		38	38	25	27	27	24	23	19	6	20	14	17	20	3
Micronesia	WPR: LIC	2019-23	1		60	40	0	23	46	14	18	22	15	15	0	25	22	0
Philippine	WPR: LIC	2019-23	2		50	38	13	26	39	15	19	30	2	17	7	21	23	0
Singapore	WPR: HIC	2017-	1		39	33	28	14	36	18	32	23	15	18	10	19	16	0

Score Card				
AMR One Health Sector	0-17%	18-35%	36-53%	54-72%
Pillars	0-11%	12-23%	24-35%	36-48%
Themes	0-7%	8-15%	16-23%	24-32%

Water Type													
Green Water				Blue Water			Brown Water						
Surface Water	Marine Water	Groundwater	Karst Groundwater	Municipal Water	Well Water	Self-Supplied Water	Agricultural Wastewater	Industrial Wastewater	Pharmaceutical Wastewater	Municipal Wastewater	Hospital Wastewater	Stormwater	

* This is India's first NAP, but the country established the National Policy on AMR Containment in 2011, which set the stage for the development of their NAP
