# Supplementary material for: Antimicrobial Resistance and Environmental Health: A Water Stewardship Framework for Global and National Action
Source: Antibiotics (Basel). 2022 Jan 5;11(1):63. doi: 10.3390/antibiotics11010063 (PMC8773023; doi:10.3390/antibiotics11010063)
Supplement: Supplementary file 1 [file antibiotics-11-00063-s001.zip › Figure S2_Linear Regression Results.pdf]

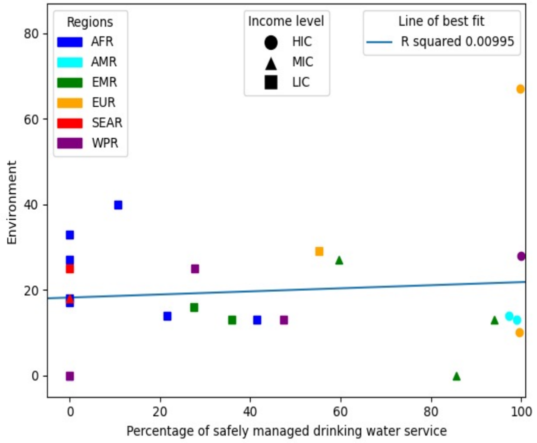

A) Linear regression between the percentage of countries reporting safely managed supply for SDG 6.1.1 and One Health: Environment and Water sector results of the National Action Plan framework evaluation.

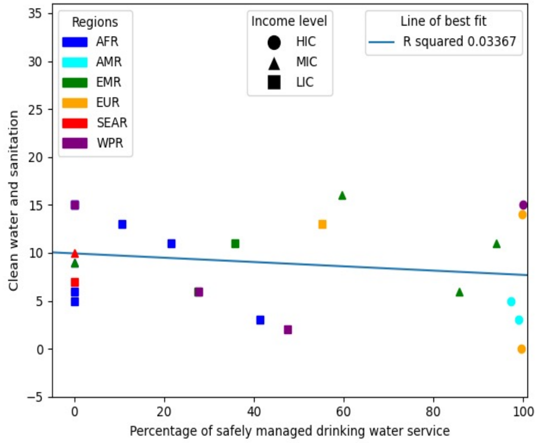

B) Linear regression between the percentage of countries reporting safely managed supply for SDG 6.1.1 and Clean Water and Sanitation theme results of the National Action Plan framework evaluation.

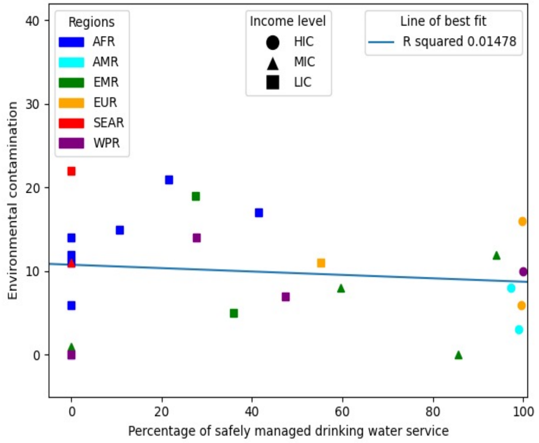

C) Linear regression between the percentage of countries reporting safely managed supply for SDG 6.1.1 and Environmental Contamination theme results of the National Action Plan framework evaluation.

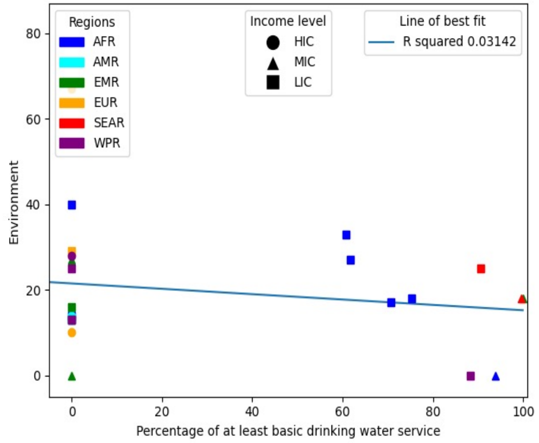

D) Linear regression between the percentage of countries reporting at least basic supply for SDG 6.1.1 and One Health: Environment and Water sector results of the National Action Plan framework evaluation.

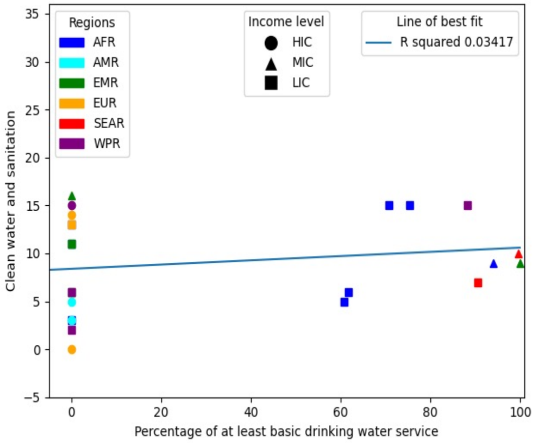

E) Linear regression between the percentage of countries reporting at least basic supply for SDG 6.1.1 and Clean Water and Sanitation theme results of the National Action Plan framework evaluation.

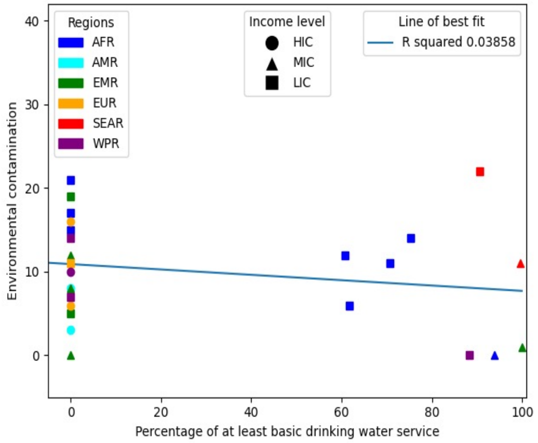

F) Linear regression between the percentage of countries reporting at least basic supply for SDG 6.1.1 and Environmental Contamination theme results of the National Action Plan framework evaluation.

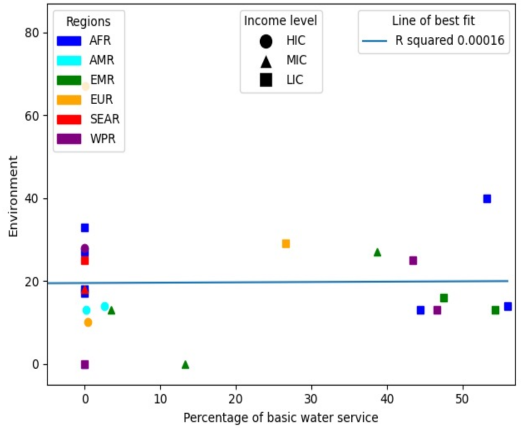

G) Linear regression between the percentage of countries reporting basic supply for SDG 6.1.1 and One Health: Environment and Water sector results of the National Action Plan framework evaluation.

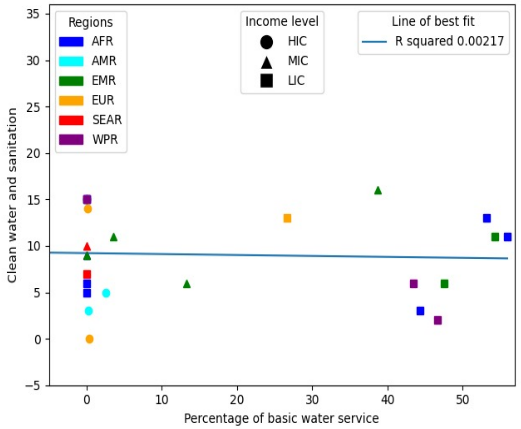

H) Linear regression between the percentage of countries reporting basic supply for SDG 6.1.1 and Clean Water and Sanitation theme results of the National Action Plan framework evaluation.

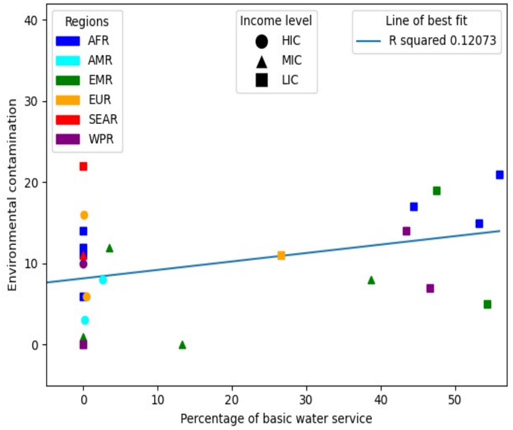

I) Linear regression between the percentage of countries reporting basic supply for SDG 6.1.1 and Environmental Contamination theme results of the National Action Plan framework evaluation.

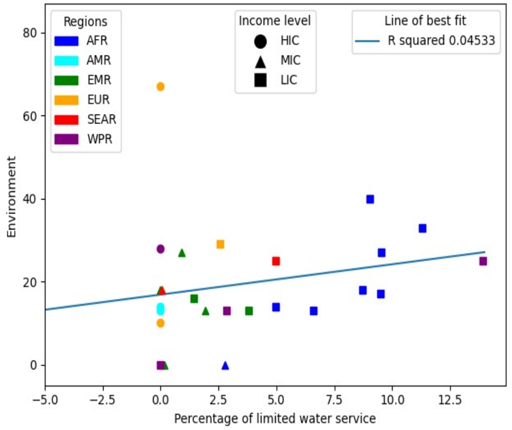

J) Linear regression between the percentage of countries reporting limited service for SDG 6.1.1 and One Health: Environment and Water sector results of the National Action Plan framework evaluation.

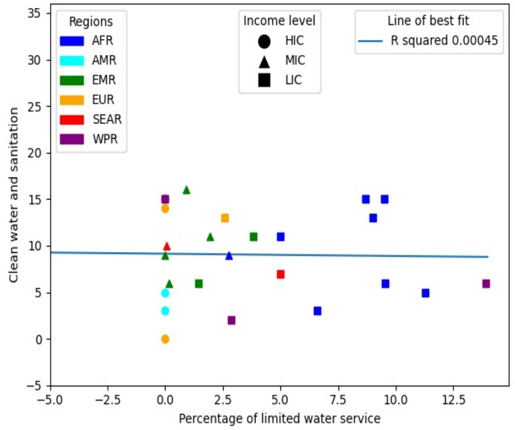

K) Linear regression between the percentage of countries reporting limited service for SDG 6.1.1 and Clean Water and Sanitation theme results of the National Action Plan framework evaluation.

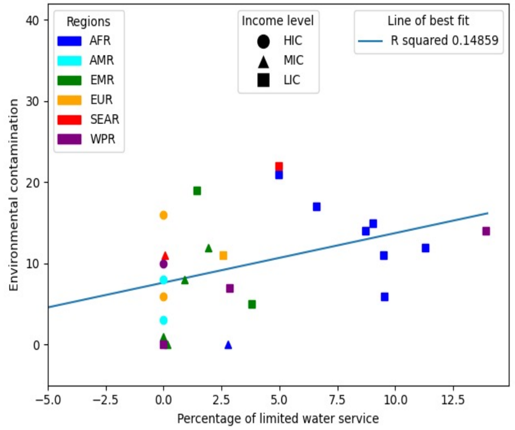

L) Linear regression between the percentage of countries reporting limited service for SDG 6.1.1 and Environmental Contamination theme results of the National Action Plan framework evaluation.

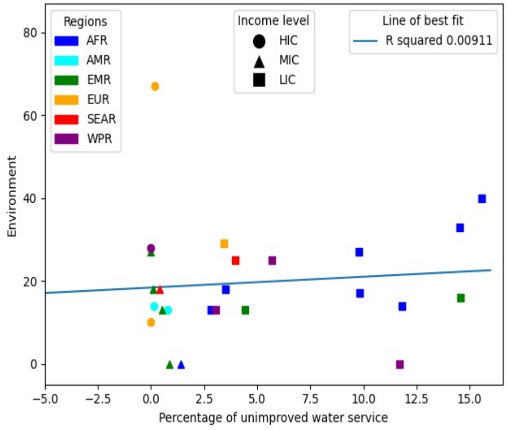

M) Linear regression between the percentage of countries reporting unimproved service for SDG 6.1.1 and One Health: Environment and Water sector results of the National Action Plan framework evaluation.

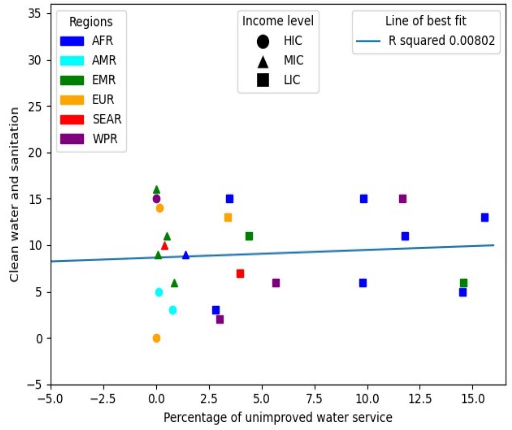

N) Linear regression between the percentage of countries reporting unimproved service for SDG 6.1.1 and Clean Water and Sanitation theme results of the National Action Plan framework evaluation.

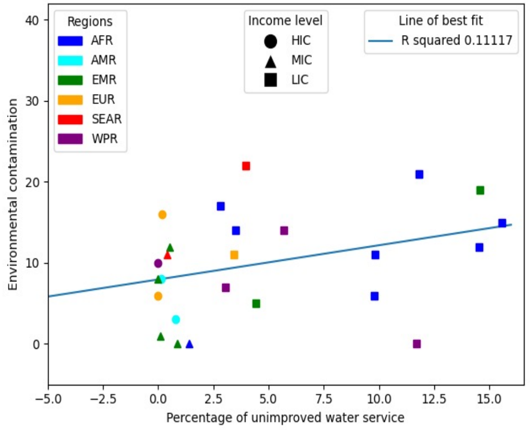

O) Linear regression between the percentage of countries reporting unimproved service for SDG 6.1.1 and the Environmental Contamination theme results of the National Action Plan framework evaluation.

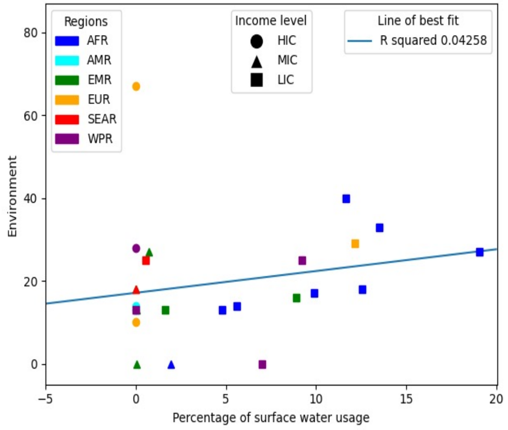

P) Linear regression between the percentage of countries reporting surface water supply for SDG 6.1.1 and One Health: Environment and Water sector results of the National Action Plan framework evaluation.

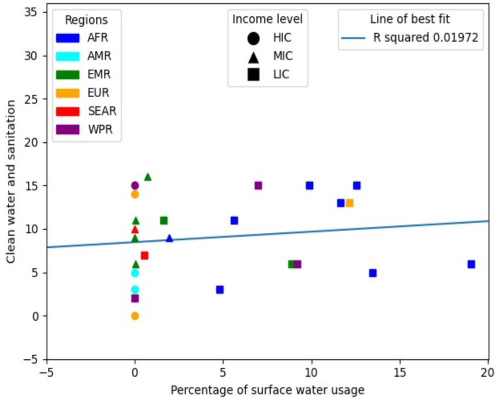

Q) Linear regression between the percentage of countries reporting surface water supply for SDG 6.1.1 and Clean Water and Sanitation theme results results of the National Action Plan framework evaluation.

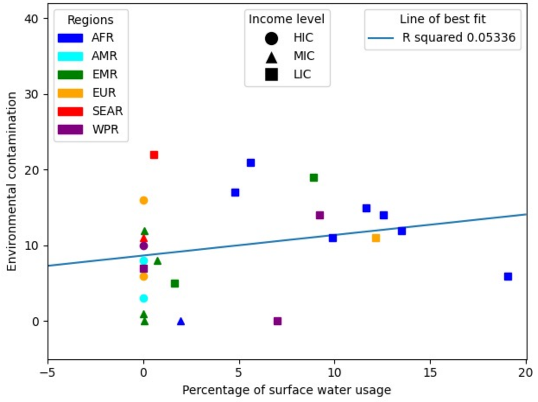

R) Linear regression between the percentage of countries reporting surface water supply for SDG 6.1.1 and the Environmental Contamination theme results of the National Action Plan framework evaluation.

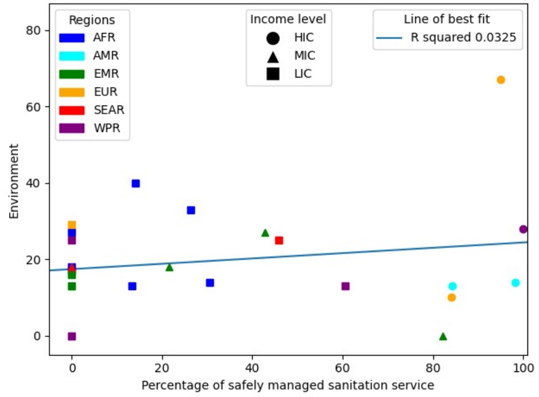

S) Linear regression between the percentage of countries reporting safely managed service for SDG 6.2.1a and One Health: Environment and Water sector results of the National Action Plan framework evaluation.

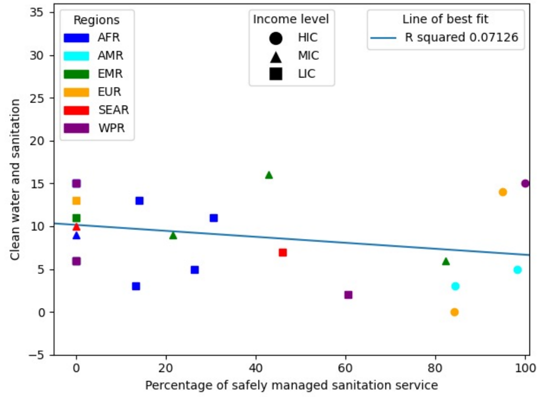

T) Linear regression between the percentage of countries reporting safely managed service for SDG 6.2.1a and Clean Water and Sanitation theme results of the National Action Plan framework evaluation.

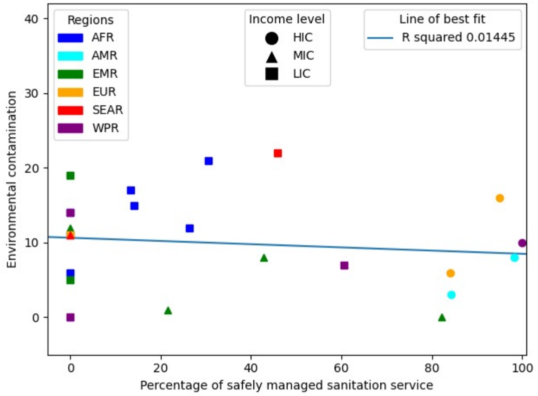

U) Linear regression between the percentage of countries reporting safely managed service for SDG 6.2.1a and Environmental Contamination theme results of the National Action Plan framework evaluation.

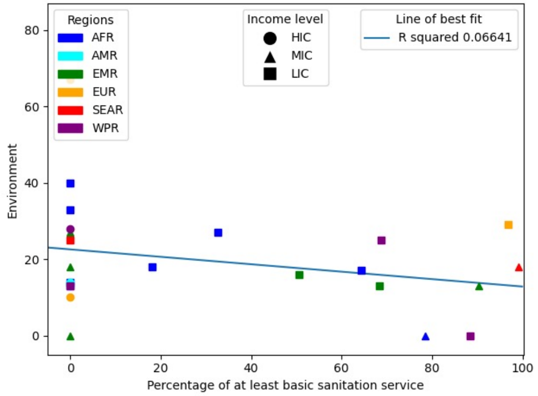

V) Linear regression between the percentage of countries reporting at least basic service for SDG 6.2.1a and One Health: Environment and Water sector results of the National Action Plan framework evaluation.

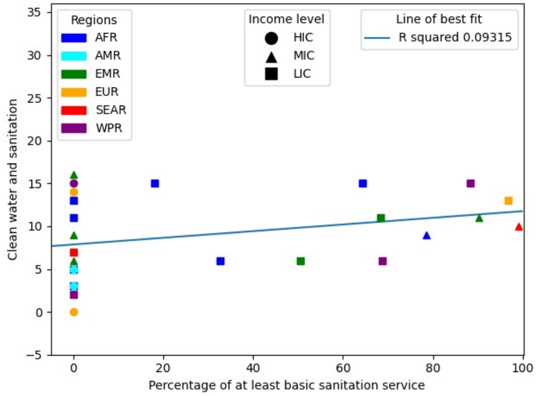

W) Linear regression between the percentage of countries reporting at least basic service for SDG 6.2.1a and Clean Water and Sanitation theme results of the National Action Plan framework evaluation.

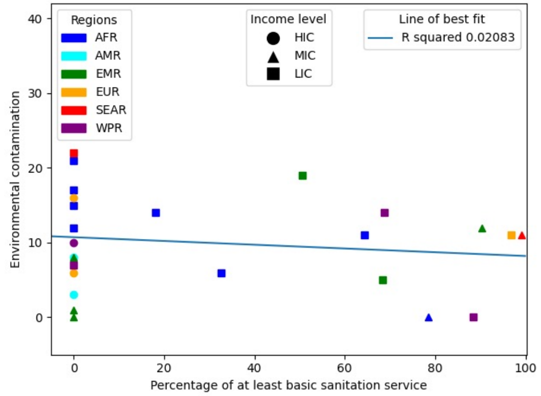

X) Linear regression between the percentage of countries reporting at least basic service for SDG 6.2.1a and Environmental Contamination theme results of the National Action Plan framework evaluation.

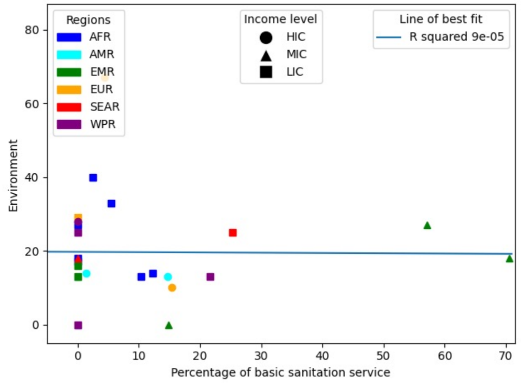

Y) Linear regression between the percentage of countries reporting basic service for SDG 6.2.1a and One Health: Environment and Water sector results of the National Action Plan framework evaluation.

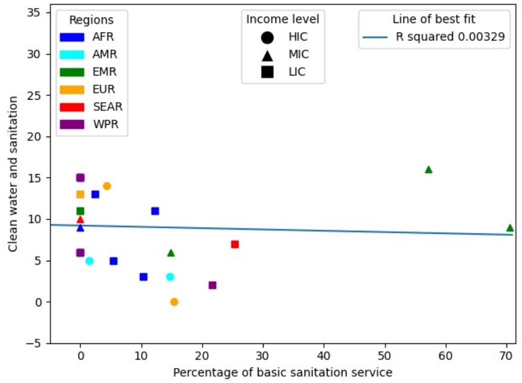

Z) Linear regression between the percentage of countries reporting basic service for SDG 6.2.1a and Clean Water and Sanitation theme results of the National Action Plan framework evaluation.

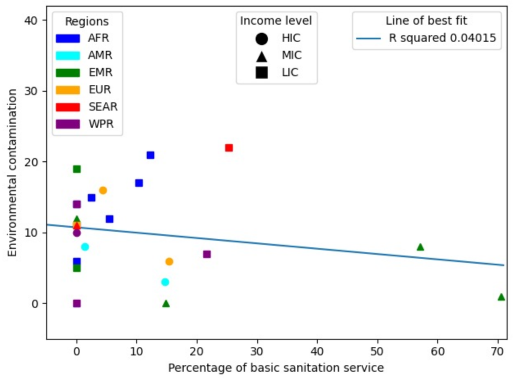

AA) Linear regression between the percentage of countries reporting basic service for SDG 6.2.1a and Environmental Contamination theme results of the National Action Plan framework evaluation.

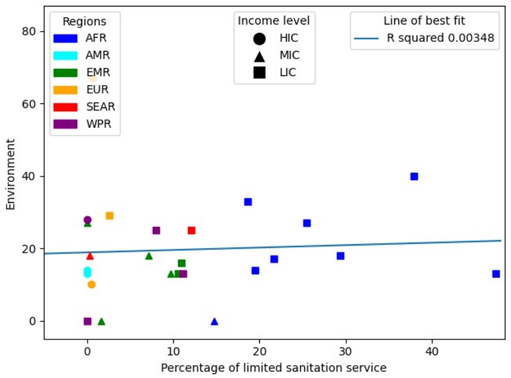

AB) Linear regression between the percentage of countries reporting limited service for SDG 6.2.1a and One Health: Environment and Water sector results of the National Action Plan framework evaluation.

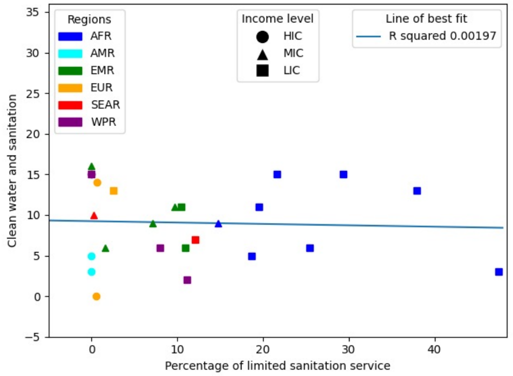

AC) Linear regression between the percentage of countries reporting limited service for SDG 6.2.1a and Clean Water and Sanitation theme results of the National Action Plan framework evaluation.

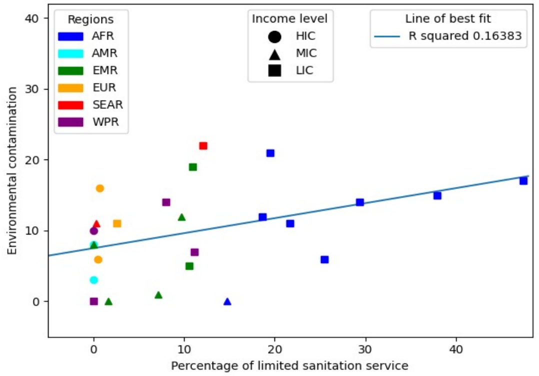

AD) Linear regression between the percentage of countries reporting limited services for SDG 6.1.2a and the Environmental Contamination theme results of the National Action Plan framework evaluation.

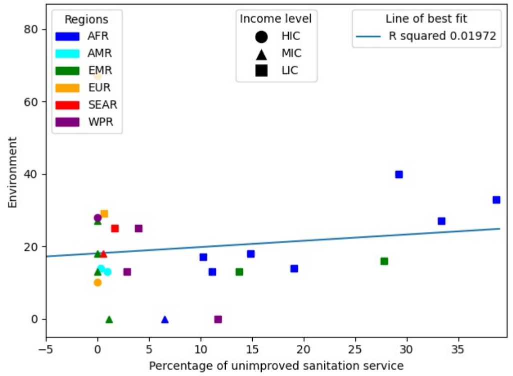

AE) Linear regression between the percentage of countries reporting unimproved service for SDG 6.2.1a and One Health: Environment and Water sector results of the National Action Plan framework evaluation.

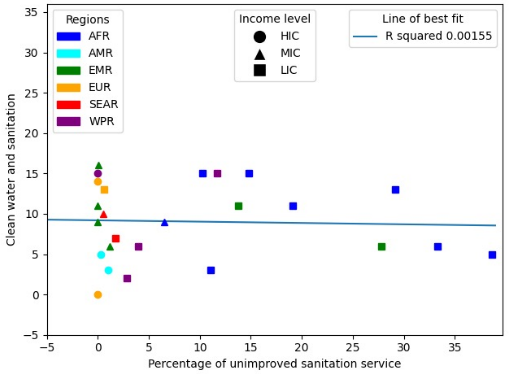

AF) Linear regression between the percentage of countries reporting unimproved service for SDG 6.2.1a and Clean Water and Sanitation theme results of the National Action Plan framework evaluation.

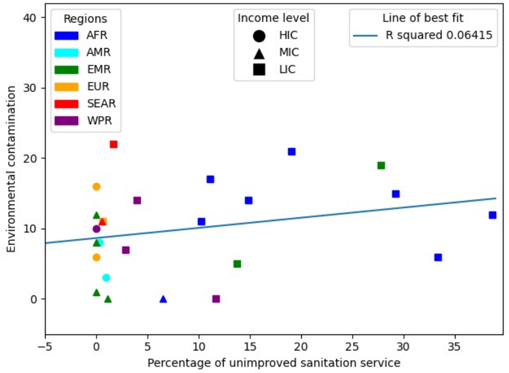

AG) Linear regression between the percentage of countries reporting unimproved service for SDG 6.2.1a Environmental Contamination theme results of the National Action Plan framework evaluation.

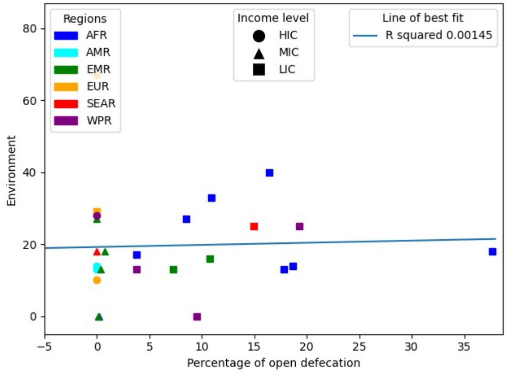

AH) Linear regression between the percentage of countries reporting open defecation in supply for SDG 6.2.1a and One Health: Environment and Water sector results of the National Action Plan framework evaluation.

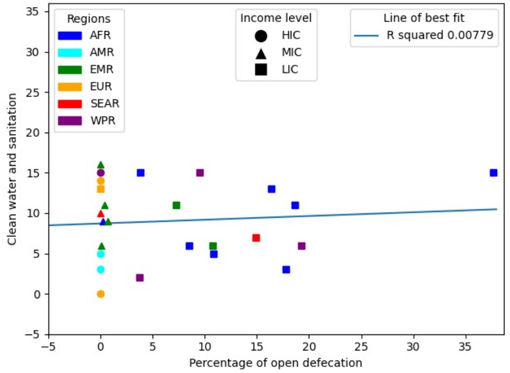

AI) Linear regression between the percentage of countries reporting open defecation in supply for SDG 6.2.1a and Clean Water and Sanitation theme results results of the National Action Plan framework evaluation.

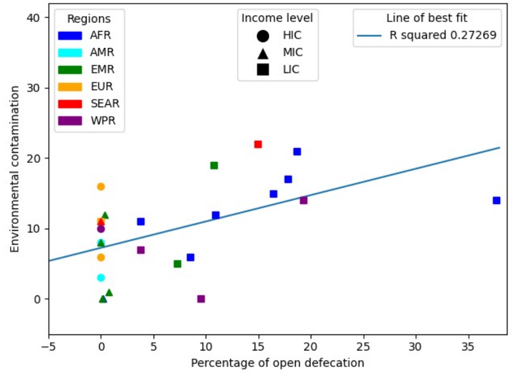

AJ) Linear regression between the percentage of countries reporting open defecation for SDG 6.1.2a and the Environmental Contamination theme results of the National Action Plan framework evaluation.

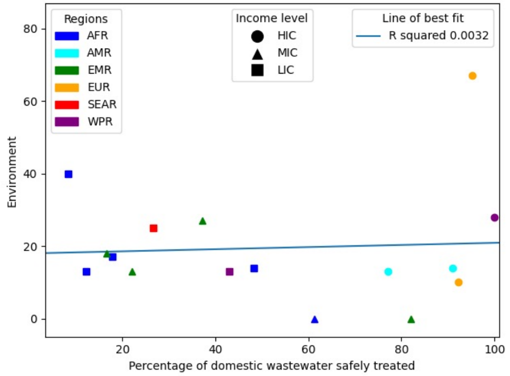

AK) Linear regression between the percentage of countries reporting safely treated domestic wastewater for SDG 6.3.1 and One Health: Environment and Water sector results of the National Action Plan framework evaluation.

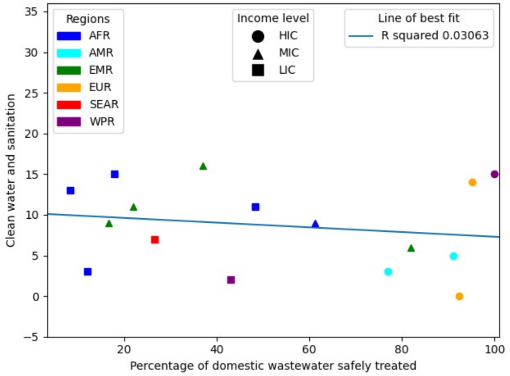

AL) Linear regression between the percentage of countries reporting safely treated domestic wastewater for SDG 6.3.1 and Clean Water and Sanitation theme results of the National Action Plan framework evaluation.

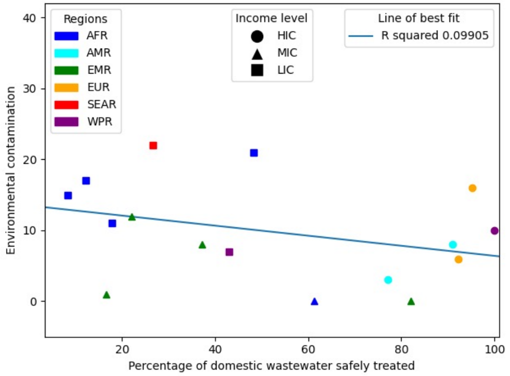

AM) Linear regression between the percentage of countries reporting safely treated domestic wastewater for SDG 6.3.1 and Environmental Contamination theme results of the National Action Plan framework evaluation.

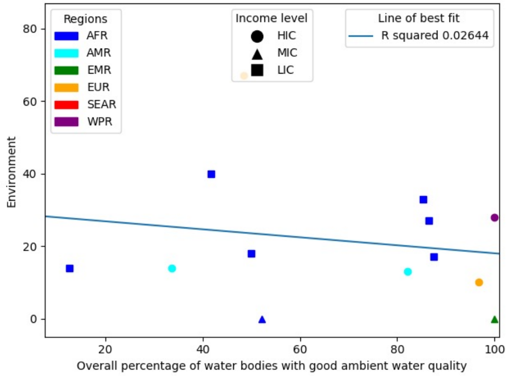

AN) Linear regression between the percentage of countries reporting good ambient water quality: overall for SDG 6.3.2 and One Health: Environment and Water sector results of the National Action Plan framework evaluation.

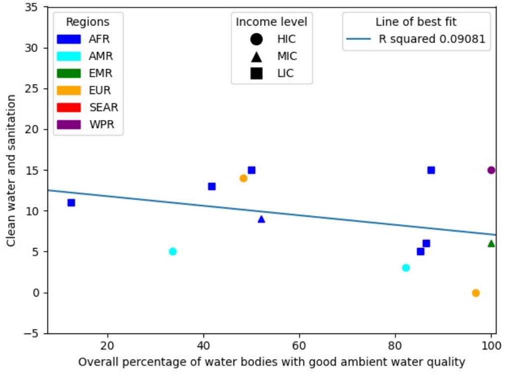

AO) Linear regression between the percentage of countries reporting good ambient water quality: overall for SDG 6.3.2 and Clean Water and Sanitation theme results of the National Action Plan framework evaluation.

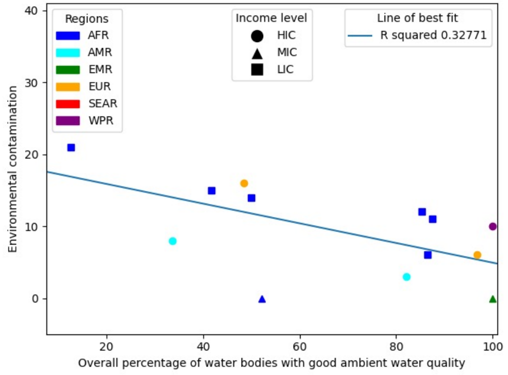

AP) Linear regression between the percentage of countries reporting good ambient water quality: overall for SDG 6.3.2 and Environmental Contamination theme results of the National Action Plan framework evaluation.

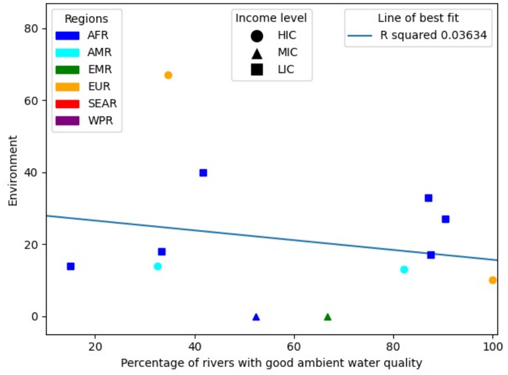

AQ) Linear regression between the percentage of countries reporting good ambient water quality: rivers for SDG 6.3.2 and One Health: Environment and Water sector results of the National Action Plan framework evaluation.

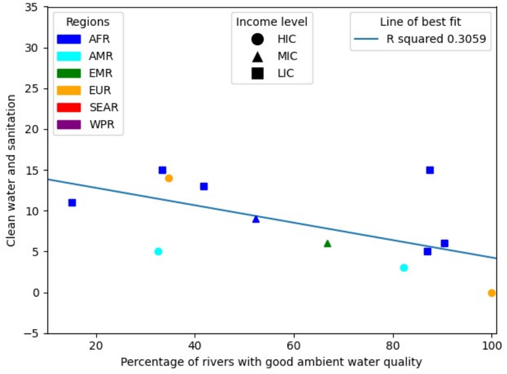

AR) Linear regression between the percentage of countries reporting good ambient water quality: rivers for SDG 6.3.2 and Clean Water and Sanitation theme results of the National Action Plan framework evaluation.

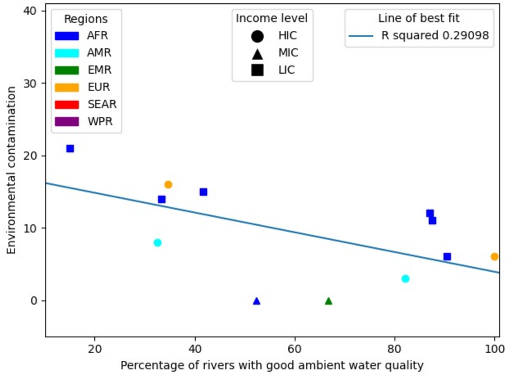

AS) Linear regression between the percentage of countries reporting good ambient water quality: rivers for SDG 6.3.2 and Environmental Contamination theme results of the National Action Plan framework evaluation.

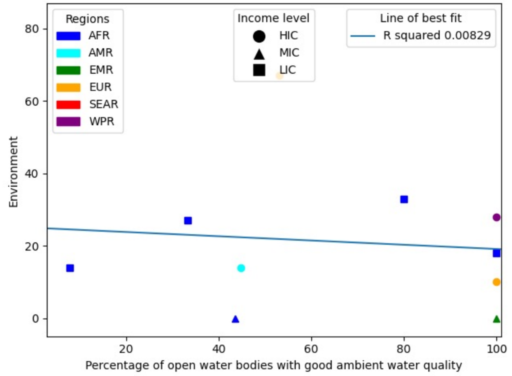

AT) Linear regression between the percentage of countries reporting good ambient water quality: open water bodies for SDG 6.3.2 and One Health: Environment and Water sector results of the National Action Plan framework evaluation.

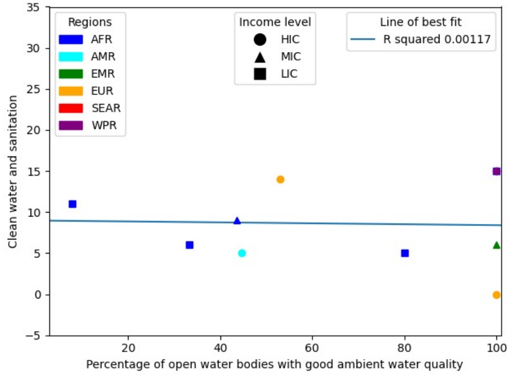

AU) Linear regression between the percentage of countries reporting good ambient water quality: open water bodies for SDG 6.3.2 and Clean Water and Sanitation theme results of the National Action Plan framework evaluation.

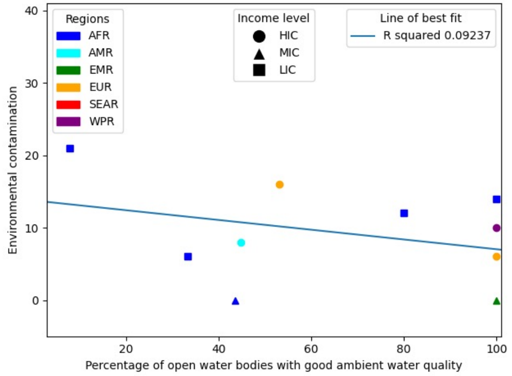

AV) Linear regression between the percentage of countries reporting good ambient water quality: open water bodies for SDG 6.3.2 and Environmental Contamination theme results of the National Action Plan framework evaluation.
