# Supplementary material for: Antimicrobial Resistance and Environmental Health: A Water Stewardship Framework for Global and National Action
Source: Antibiotics (Basel). 2022 Jan 5;11(1):63. doi: 10.3390/antibiotics11010063 (PMC8773023; doi:10.3390/antibiotics11010063)
Supplement: Supplementary file 1 [file antibiotics-11-00063-s001.zip › Table S1_NAP-CaseStudy-Parameters.pdf]

Country	WHO Region & World Bank Country Income Classification	NAP Timeframe (Year)	NAP Version
Eswatini	AFR: LIC	2018-22	1
Ghana	AFR: LIC	2017-21	2
Kenya	AFR: LIC	2017-22	1
Liberia	AFR: LIC	2018-22	1
Nigeria	AFR: LIC	2017-22	1
Sierra Leone	AFR: LIC	2018-22	1
South Africa	AFR: MIC	2018-24	2
Tanzania	AFR: LIC	2017-22	1
Canada	AMR: HIC	2017- **	1
United States	AMR: HIC	2020-25	2
Afghanistan	EMR: LIC	2017-21	1
Iran	EMR: MIC	2016-21	1
Iraq	EMR: MIC	2018-22	1
Jordan	EMR: MIC	2018-22	1
Libya	EMR: MIC	2019-23	1
Pakistan	EMR: LIC	2017- **	1
Finland	EUR: HIC	2017-21	1
Sweden	EUR: HIC	2020-23	2
Tajikistan	EUR: LIC	2018- **	1
India	SEAR: LIC	2017-21	2*
Maldives	SEAR: MIC	2017-22	1
Cambodia	WPR: LIC	2019-23	2
Micronesia	WPR: LIC	2019-23	1
Philippine	WPR: LIC	2019-23	2
Singapore	WPR: HIC	2017- **	1

* This is India's first NAP, but the country established the National Policy on AMR Containment in 2011, which set the stage for the development of their NAP
** No end date was noted for the NAP
