# Supplementary material for: Antimicrobial Resistance and Environmental Health: A Water Stewardship Framework for Global and National Action
Source: Antibiotics (Basel). 2022 Jan 5;11(1):63. doi: 10.3390/antibiotics11010063 (PMC8773023; doi:10.3390/antibiotics11010063)
Supplement: Supplementary file 1 [file antibiotics-11-00063-s001.zip › Table S2_Framework_Guidelines.pdf]

AMR Stewardship Theme	Definitions (modified from Wellcome Trust 2020)	Keywords
Human Infection Prevention and Control	This theme focuses on preventing the spread of human infection in healthcare settings through effective control practices, including handwashing and PPE.	Infection Prevention and Control, hygiene, hand hygiene, personal protective equipment, PPE, disinfection, sterilization, safe disposal, environmental cleaning, isolation, hospital/clinical waste management, sanitation, surfaces, stewardship, hospital, clinical, health care facility
Clean Water and Sanitation	This theme focuses on clean water, sanitation, and hygiene (WASH) access and safe wastewater disposal to reduce the environmental spread of pathogens and AMR organisms, as well as reduce the need for antimicrobial use for WASH-related illnesses..	clean water, sanitation, hygiene, WASH, water supply, intervention, leaching, leachate, reservoir, wastewater, effluent, influent, surface water, lake, stream, marine, groundwater, karst groundwater, drinking water, source water, water supply, fecal matter, treatment, filtration, chlorination, removal
Food Safety and Security	This theme is focused on food safety and security across the food chain (i.e., from farm to table) of soils, plants, and animals that can expose humans to AMR organisms through food handling or consumption. .	food production, food supply, food safety, food security, food handling, food processing, food contamination, food retail, food packaging, food import, food export, food system, grocery, foodborne, food surfaces
Environmental Contamination	This theme is focused on how environmental systems – principally soil, water and air – may expose to human to AMR organisms through three primary mechanisms:<br>1. Environment as a transmission vector.<br>2. Environment introducing selective pressures (heavy metals, antibiotics, etc.) for the	environmental contamination, environmental AMR, pollution, source of resistance, run-off, wildlife, ecology, ecosystem, remediation, soil contamination

	development of AMR through pollutant mixing<br>3. Environment as a reservoir of novel ARG.<br>Overall this theme focuses on the transmission of AMR from environmental systems to humans and potential innovations to reduce impacts.	
Human Consumption of Antimicrobials	This theme focuses on the prescription, dispensing, misuse, improper dosage, improper diagnosis, and consumption of antimicrobials within humans, which is increasing the risk of available antimicrobials being ineffective.	consumption, antibiotic overuse, antibiotic prescribing, antibiotic use, antibiotic access, over the counter, antibiotic misuse, dosage, treatment duration, daily dose, prescribing practices, stewardship, hospital, clinical, health care facility
Use of Antimicrobials in Animals	This theme is focused on the use of antimicrobials in food animal production. Antimicrobials are used for animal well-being, disease prevention, growth promotion, or prophylactic group treatment. The main concern within this theme is the unnecessary and overuse of antimicrobials, which are then introduced to the natural environment or humans directly.	farm animals, food producing animals, antibiotics in animals, livestock, veterinary, companion animals, husbandry, probiotic, zoonosis, aquaculture
Use of Antimicrobials in Plants	The focus of this theme is the utilization of antimicrobials in food plant production. Food plant products are exposed to antimicrobials from direct application of mass crop spray such as manure fertilizers. The food plants are able to be infected with resistant pathogens and directly introduced to humans through consumption. This area of agriculture has received little attention in comparison to food animal production, but may be a significant reservoir of AMR.	plants, agriculture, pesticides, herbicides, fungicides, fertilizers, horticulture, botany, fruit, vegetable, harvest, crop, nursery, biocide, leaf, legume, roots, fresh produce
