# Supplementary material for: Antimicrobial Resistance and Environmental Health: A Water Stewardship Framework for Global and National Action
Source: Antibiotics (Basel). 2022 Jan 5;11(1):63. doi: 10.3390/antibiotics11010063 (PMC8773023; doi:10.3390/antibiotics11010063)
Supplement: Supplementary file 1 [file antibiotics-11-00063-s001.zip › Table S3_Framework_Scoring_Guidelines.pdf]

Response:	Criteria:	Weighted Score:	Scoring Guidelines	Water focus Scoring Guidelines
**A**	Not included in NAP	0	The sub lever in relation to the designated theme was not mentioned within the NAP	In relation to water focus
**B**	Acknowledged, but no plan of action in NAP	0.25	The sub lever in relation to the designated theme was mentioned within the NAP but no concrete steps, goals, or plans were outlined	In relation to water focus
**C**	Plan of action outlined in NAP	0.5	The sub lever in relation to the designated theme was mentioned within the NAP and goals, timeline, future steps, plans were outlined	In relation to water focus
**D**	Implementation in NAP	0.75	The sub lever in relation to the designated theme was mentioned within the NAP and a plan of action and program was implemented	In relation to water focus
**E**	Established active plan of action/ stewardship program in NAP	1	The sub lever in relation to the designated theme was mentioned within the NAP and an established active plan and program are continuously implemented and evaluated	In relation to water focus
**NA**	Not applicable			
